# Inducible In Vivo Silencing of Brd4 Identifies Potential Toxicities of Sustained BET Protein Inhibition

**DOI:** 10.1016/j.celrep.2014.08.025

**Published:** 2014-09-18

**Authors:** Jessica E. Bolden, Nilgun Tasdemir, Lukas E. Dow, Johan H. van Es, John E. Wilkinson, Zhen Zhao, Hans Clevers, Scott W. Lowe

**Affiliations:** 1 Memorial Sloan-Kettering Cancer Center, New York, NY 10065, USA; 2 Watson School of Biological Sciences, Cold Spring Harbor, NY 11724, USA; 3 Cold Spring Harbor Laboratory, Cold Spring Harbor, NY 11724, USA; 4 Hubrecht Institute/KNAW, Uppsalalaan 8, Utrecht 3584 CT, the Netherlands; 5 Department of Pathology, University of Michigan School of Medicine, Ann Arbor, MI 48109, USA; 6 University Medical Center Utrecht, Uppsalalaan 8, Utrecht 3584 CT, the Netherlands; 7 Howard Hughes Medical Institute, New York, NY 10065, USA

## Abstract

BET family proteins are novel therapeutic targets for cancer and inflammation and represent the first chromatin readers against which small-molecule inhibitors have been developed. First-generation BET inhibitors have shown therapeutic efficacy in preclinical models, but the consequences of sustained BET protein inhibition in normal tissues remain poorly characterized. Using an inducible and reversible transgenic RNAi mouse model, we show that strong suppression of the BET protein Brd4 in adult animals has dramatic effects in multiple tissues. Brd4-depleted mice display reversible epidermal hyperplasia, alopecia, and decreased cellular diversity and stem cell depletion in the small intestine. Furthermore, Brd4-suppressed intestines are sensitive to organ stress and show impaired regeneration following irradiation, suggesting that concurrent Brd4 suppression and certain cytotoxic therapies may induce undesirable synergistic effects. These findings provide important insight into Brd4 function in normal tissues and, importantly, predict several potential outcomes associated with potent and sustained BET protein inhibition.

## INTRODUCTION

The *b*romodomain and extra*t*erminal (BET) family of chromatin reader proteins are an exciting new class of therapeutic targets in inflammation and in solid and hematopoietic cancers. Development and optimization of small molecules that antagonize BET activity (Filippakopoulos et al., 2010; Nicodeme et al., 2010) has led to the initiation of phase 1/2 clinical trials, yet we know surprisingly little about the consequences of disrupting BET protein function in normal tissues. Brd4 is one of four mammalian BET family members, which, through its dual bromodomains, recognizes and binds acetyl-lysine residues of histone and nonhistone proteins (Wu and Chiang 2007). Brd4 serves as a chromatin scaffold, enabling the recruitment of transcription factors, transcriptional coactivators and corepressors, and core transcriptional machinery to acetylated target gene promoters (Belkina and Denis 2012). Brd4 thereby regulates diverse biological processes, including the cell cycle (Dey et al., 2009), inflammation (Huang et al., 2009; Nicodeme et al., 2010), maintenance of higher-order chromatin structure (Wang et al., 2012), and DNA damage signaling (Floyd et al., 2013).

An immediate rationale for the development of BET/Brd4 inhibitors was provided by the discovery of recurrent t(15;19) chromosomal translocations (and the resulting in-frame fusion of Brd4 and nuclear protein in testis [NUT]) as the cause of NUT midline carcinoma (NMC) (Filippakopoulos et al., 2010; French et al., 2001). The subsequent identification of *Brd4* as an important maintenance gene in acute myeloid leukemia (AML) fueled further interest in Brd4 as a cancer target (Zuber et al., 2011b). First-generation synthetic BET inhibitors, such as JQ1, mimic acetylated-lysine moieties and bind to the bromodomain pockets of all four BET family members (Brd2, Brd3, Brd4, and BrdT) (Filippakopoulos et al., 2010, Nicodeme et al., 2010). Such inhibitors have demonstrated anticancer activity in preclinical murine and xenograft models of NMC, AML, multiple myeloma, and Burkitt’s lymphoma (Delmore et al., 2011; Filippakopoulos et al., 2010; Mertz et al., 2011; Zuber et al., 2011b), and their derivatives have entered phase 1/2 clinical trials for the treatment of NMC and hematological malignancies (Mirguet et al., 2013).

In AML, BET inhibitors are thought to act largely by inhibiting the expression of c-Myc and, consequently, block the self-renewal and survival of leukemia cells (Delmore et al., 2011; Grayson et al., 2014; Mertz et al., 2011; Zuber et al., 2011b). As such, these agents provide a unique opportunity to inhibit this undruggable transcription factor. However, in nonhematological cancers and noncancer pathologies (i.e., inflammation), BET inhibitors appear to suppress the function of other transcriptions such as FOSL1 and NF-κB (Huang et al., 2009; Lockwood et al., 2012). Although less is known about BET action in normal tissues, mice null for *Brd4* die early after implantation and heterozygote *Brd4*^+/−^ mice exhibit higher rates of postnatal death, reduced growth rates, and a variety of serious developmental abnormalities (Houzelstein et al., 2002). While these observations raise toxicity concerns, preclinical tests with first-generation pan-BET inhibitors indicate that mice can tolerate therapeutic doses of JQ1 for up to 3 months (Matzuk et al., 2012). However, the pharmacokinetic properties of the first-generation compounds did not allow sustained target inhibition and thus showed limited efficacy. Therefore, the full range of potential toxicities that might be encountered upon achieving sustained target inhibition has not yet been reported.

Our laboratory recently described a platform for the production of transgenic mice harboring tetracycline/doxycycline (dox)-responsive short hairpin RNAs (shRNAs) that allow temporal and spatial control of endogenous gene expression. Importantly, since shRNAs do not modify the genomic loci of target genes, the system enables transient, reversible gene silencing (Dow et al., 2012; Premsrirut et al., 2011), thereby facilitating genetic loss-of-function studies as well as an assessment of reversible and irreversible phenotypes simply by adding and removing dox from the diet. When applied to candidate drug targets, such models can point toward potential toxicities associated with on-target gene inhibition, determine the kinetics of their appearance precisely from target knockdown, and evaluate whether any or all are reversible. Here, we used this approach to identify consequences of Brd4 suppression in adult tissues and observed significant yet reversible deleterious phenotypes that should inform clinical use of BET inhibitors.

## RESULTS

### Brd4 Suppression Alters Normal Hematopoiesis

**Figure 1:**
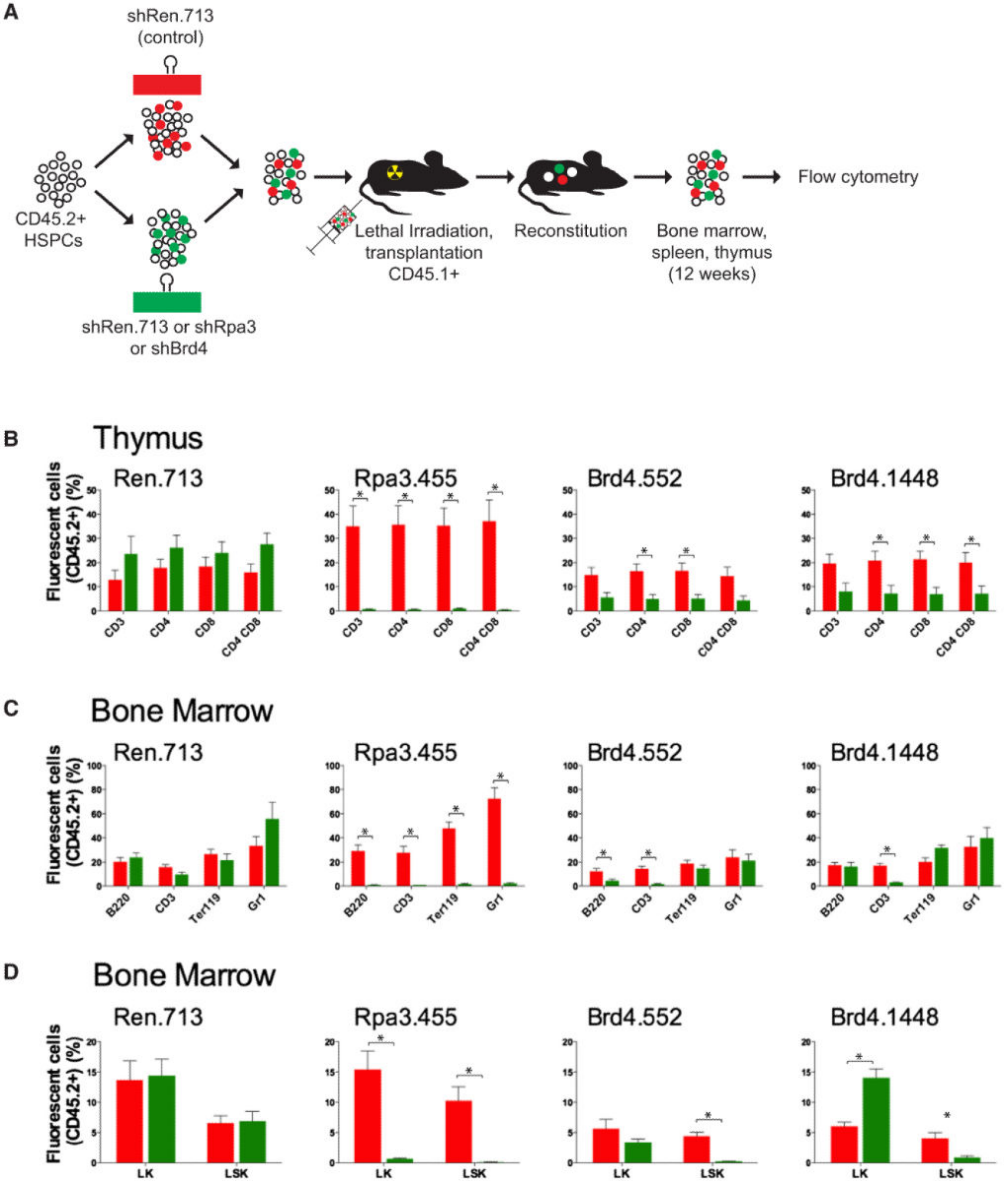
Brd4 Knockdown Affects Normal Hematopoiesis (A) Schematic representation the of reconstitution assay. Hematopoietic stem and progenitor cells (HSPCs) from CD45.2^+^ mice were retrovirally transduced to express a neutral control shRNA against Renilla luciferase (shRen.713-Cherry^+^) or an experimental shRNA (GFP^+^). Infected populations were mixed at 1:1 ratio and transplanted into lethally irradiated CD45.1^+^ recipients. Hematopoietic lineages within the spleen, thymus, and bone marrow were examined for the presence of Cherry^+^ and GFP^+^ donor-derived cells 12 weeks posttransplantation. An shRNA against replication protein A3 (shRpa3.455) serves as a strong positive control for depletion. Two independent shRNAs targeting Brd4 (shBrd4.1448 and shBrd4.552) were used. (B–D) Thymus (B), bone marrow lineage (C), and bone marrow stem cell composition (D). The percentage of CD45.2^+^ cells expressing shRen.713 (red) and the indicated experimental hairpin (green) in specific hematopoietic lineages (B220+ B cells, CD3+ T cells and CD4/CD8 T cell subsets, Ter119+ erythroid cells and Gr1+ granulocytes) are shown. In addition, myeloid progenitors (LK: Lineage^−^, cKit^+^, Sca1^−^) and hematopoietic stem cells (LSK: Lineage^−^, cKit^+^, Sca1^+^) are shown. Data are presented as mean + SEM (n = 4). Asterisks (*) indicate a statistically significant difference between the presence of neutral control and experimental shRNAs (p < 0.05), as determined by a two-tailed Student’s t test.

BET inhibitors are entering phase 1/2 clinical trials for the treatment of hematological malignancies, yet little is known about how they impact normal hematopoietic development. To examine the consequence of Brd4 suppression on normal hematopoiesis, we performed a two-color competitive reconstitution assay, which measures the in vivo “fitness’ of cells harboring experimental shRNAs (marked by GFP fluorescence) against cells expressing a neutral shRNA targeting Renilla luciferase (marked by mCherry) (Figure 1A) (Zuber et al., 2011a). To minimize the possibility that any effects could be due to “off-target’ silencing, we performed reconstitutions with two independent Brd4 shRNAs (shBrd4.1448 and shBrd4.552) (Figure S1A), previously shown to potently inhibit Brd4 expression in AML cells (Zuber et al., 2011b). Importantly, in vitro RNAi-mediated silencing of Brd4 produces a gene expression profile similar to that of treatment with the BET inhibitor JQ1, suggesting that RNAi is a suitable surrogate for Brd4-targeted drugs (Zuber et al., 2011b).

Twelve weeks following hematopoietic reconstitution, we measured GFP^+^ and mCherry^+^ cells within specific hematopoietic subsets in the bone marrow, thymus, and spleen of recipient mice. Although the Brd4.552 hairpin generally performs more strongly in in vitro mouse embryonic fibroblast (MEF) depletion assays (Zuber et al., 2011b), both shRNAs caused similar levels of Brd4 knockdown in retrovirally transduced stem and progenitor cells (Figure S1A), and thus we focused our analysis on statistically significant changes observed with both shRNAs. In all tissues analyzed, Brd4 silencing caused depletion of T lymphoid cells, including CD4^+^ and CD8^+^ single-positive T-lineage subsets (Figures 1B, 1C, and S1B). Both shRNAs also caused a significant reduction in Lineage^−^ Sca1^+^ cKit^+^ hematopoietic stem cells in reconstituted bone marrow (Figure 1D). Thus, in addition to its demonstrated activity against hematopoietic malignancies, sustained Brd4 suppression can adversely influence normal hematopoiesis.

### Construction of shBrd4 Transgenic Mice

To further explore the consequence of Brd4 suppression on normal (nonhematopoietic) tissues, we developed Brd4-targeted shRNA transgenic strains, which enable the inducible and reversible silencing of Brd4 in vivo. Our laboratory has previously reported the generation of transgenic shRNA mice using recombinase-mediated cassette exchange that direct single-copy transgenic integrations downstream of the Collagen type I gene (*Col1a1*) (Dow et al., 2012; Premsrirut et al., 2011). Using this approach, we generated transgenic mice carrying Brd4.552, Brd4.1448, or control Ren.713 shRNAs, linked to GFP, under the control of the TRE-tight promoter (hereafter referred to as TtG-Brd4.552, TtG-Brd4.1448, and TtG-Ren.713).

**Figure 2:**
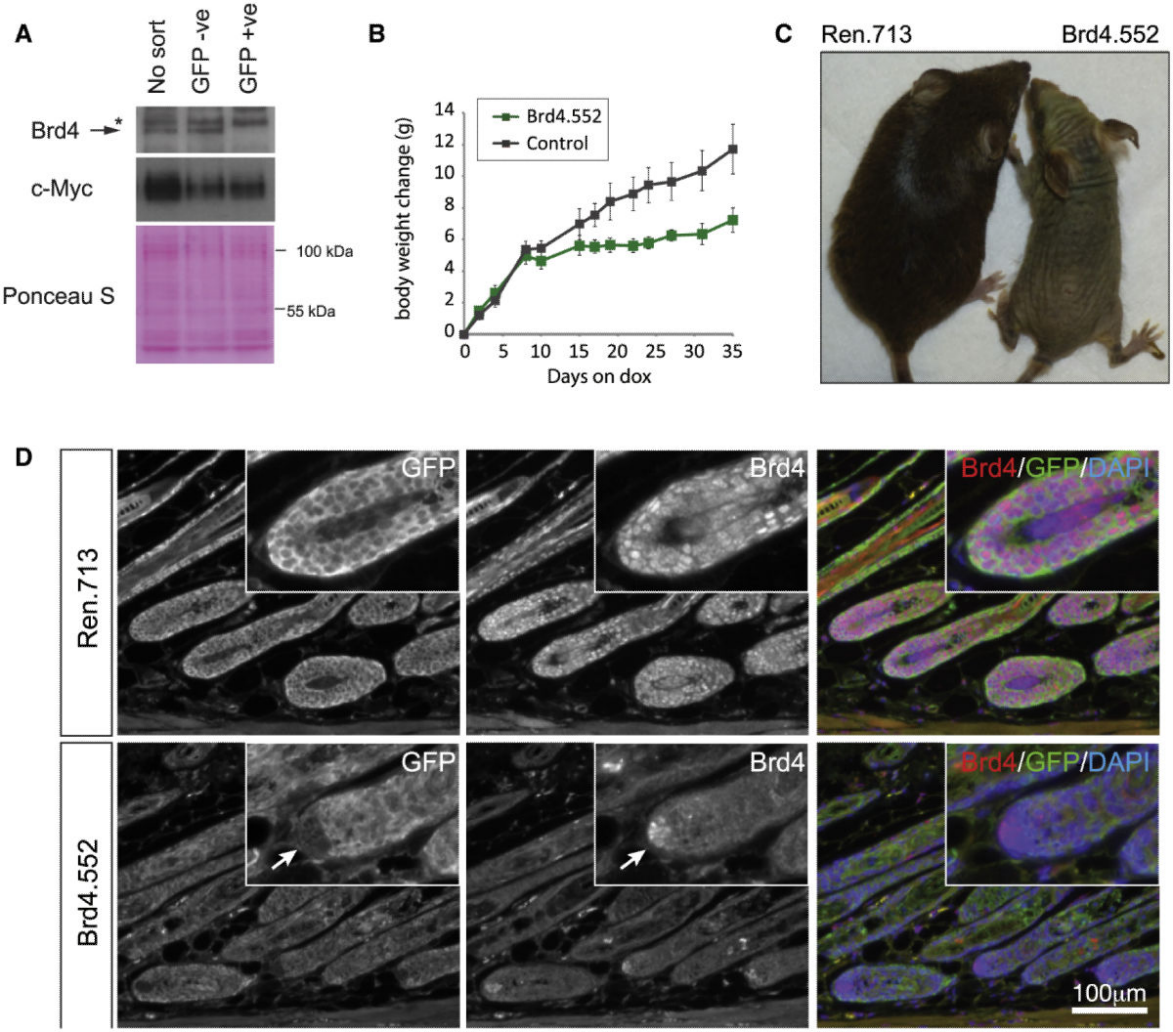
Characterization of Transgenic shBrd4 Mice (A) Immunoblot for Brd4 and c-Myc expression in nuclear extracts from R26^rtTA/+^; TtG-shBrd4.552 MEFs treated for 4 days with doxycycline (1 μg/ml). MEFs were sorted on the basis of GFP expression or left unsorted. Asterisk (*) denotes a nonspecific band detected in MEF extracts. Ponceau S stains indicate protein loading. (B) Mean weight changes (g) of male and female (combined) CAG^rtTA3/+^; TtG-Brd4.552 mice on the dox diet, relative to day 0 of dox treatment. Littermate controls include double-transgenic CAG^rtTA3/+^; TtG-Ren.713 mice and single-transgenic mice that carry a TtG-shRNA but lack a tet-transactivator. Error bars represent SEM (n = 6). (C) Image of a CAG^rtTA3/+^; TtG-Brd4.552 and littermate control CAG^rtTA3/+^; TtG-Ren.713 mouse treated with doxycycline for 5 weeks. (D) Immunofluorescence analysis of GFP and Brd4 in dorsal skin sections from CAG^rtTA3^; TtG-shRNA mice. Arrows indicates the dermal papilla, where shRNAs fail to be expressed (GFP negative) and Brd4 expression is thus retained in CAG^rtTA3/+^; TtG-Brd4 mice.

To validate Brd4 knockdown in this system, double-transgenic *R26*-rtTA; TtG-Brd4.552 mouse embryo fibroblasts were generated and cultured in the presence of dox for 4 days. Brd4 western blots on nuclear extracts from unsorted and sorted GFP^+^ and GFP^−^ cells showed a marked reduction of Brd4 protein in only GFP^+^ (shBrd4-expressing) cells (Figure 2A). Brd4 silencing in AML cells causes suppression of c-Myc expression (Zuber et al., 2011b); however, despite potent knockdown, we did not observe any changes in Myc protein levels following Brd4 knockdown in MEFs (Figure 2A). Similarly, substantial in vivo Brd4 knockdown was observed in thymus extracts from mice fed the dox diet for 2 weeks. Likewise, in thymus tissue, basal Myc protein levels remained largely unchanged (Figure S2A). Collectively, these results indicate that shBrd4 transgenes can effectively suppress Brd4 protein in vitro and in vivo and that Brd4 is not invariably required for Myc expression.

Next, we examined the consequences of sustained Brd4 knockdown in adult tissues by initiating Brd4 silencing in 3- to 5-week-old mice. *R26*-rtTA; TtG-Brd4 animals did not display any overt phenotype following 4 weeks of doxycycline treatment despite uniform GFP (and Brd4 shRNA) expression in the skin, small and large intestine, and thymus (Figure S2B). In order to increase the efficiency of Brd4 silencing, we crossed the TtG-Brd4 alleles to mice harboring a CAG-rtTA3 transgene, which produces stronger and more ubiquitous target gene knockdown (Premsrirut et al., 2011) (Figure S2C). Indeed, when double-transgenic CAG-rtTA3; TtG-shRNA mice were fed a dox diet, GFP expression was additionally observed in the pancreas, stomach, and seminal vesicles and GFP expression was substantially brighter in the skin, small intestine, and lower gastrointestinal tract (Figure S2B). Double-transgenic CAG-rtTA3; TtG-Brd4.552 mice showed a mild, but not significant, reduction in weight gain over 2 weeks compared to control CAG-rtTA3; TtG-Ren.713 mice, indicating that potent Brd4 suppression was not immediately toxic (Figures 2B and S3A), unlike the severe weight loss that has been reported in other transgenic shRNA strains targeting essential genes (McJunkin et al., 2011). CAG-rtTA3; TtG-Brd4.552 mice maintained on dox for 5 weeks showed a significant decrease in weight gain (Figure 2B), and although we did not observe the same consistent difference with the Brd4.1448 shRNA, some animals showed reduced weight gain and eventual weight loss (Figure S3A). This difference perhaps suggests that slight variations in Brd4 levels could have a significant functional impact, but it is possible that other complications arising from Brd4 depletion influenced the health of these animals.

### Brd4 Silencing Produces Skin Hyperplasia

Sustained Brd4 silencing induced striking phenotypic changes in the skin. Within 5 weeks of dox treatment, shBrd4 mice displayed follicular dysplasia characterized by abnormal hair growth and subsequent alopecia (Figures 2C and S3B), whereas control mice maintained on dox, and CAG-rtTA3; TtG-Brd4 mice maintained on a normal diet (data not shown), were phenotypically normal. Immunofluorescence analyses of skin sections from control mice showed that Brd4 was robustly expressed in the outermost layer of the inner root sheath, in the epidermis (basal and first suprabasal layer), and at the base of the hair follicle, including the dermal papillae (Figure 2D). Dox-fed CAG-rtTA3; TtG-Brd4 mice showed strong GFP expression and Brd4 silencing in the hair follicle and epidermis (Figure 2D). Of note, small regions within the dermal papilla did not express the GFP-linked shRNA and retained Brd4 expression. While it is unclear what accounts for the lack of transgene induction in these cells, it occurred in both shRen control and shBrd4 animals, suggesting a technical issue rather than active silencing of the Brd4 shRNA.

**Figure 3:**
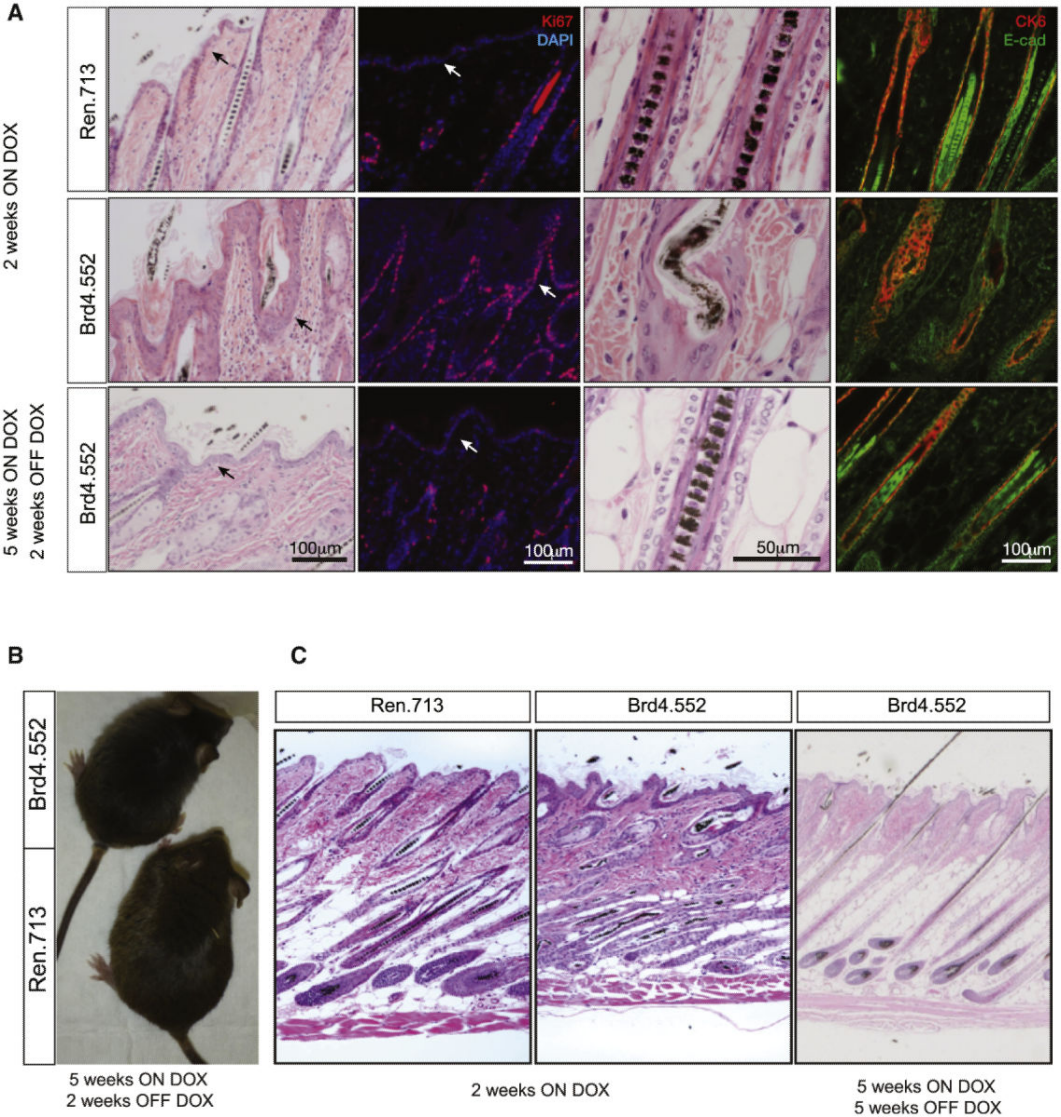
Brd4 Suppression Promotes Epithelial Hyperplasia and Follicular Defects in the Skin (A) Representative hematoxylin and eosin (H&E) and immunofluorescent stains of dorsal skin sections from CAG^rtTA3/+^; TtG-Ren.713 and CAG ^rtTA3/+^; TtG-Brd4.552 mice on the dox diet for 2 weeks, showing hair shaft defects and epithelial hyperplasia. The bottom panels show dorsal skin sections in CAG^rtTA3/+^; TtG-Brd4.552 mice following 5 weeks of dox treatment and 2 weeks of subsequent dox withdrawal. Scale bars are indicated. (B) Image of a CAG^rtTA3/+^; TtG-Brd4.552 and littermate control CAG^rtTA3/+^; TtG Ren.713 mouse following 5 weeks of doxycycline treatment and 2 weeks of subsequent dox withdrawal. (C) Full-thickness scans of H&E-stained dorsal skin sections from CAG^rtTA3/+^; TtG-Ren713 and CAG^rtTA3/+^; TtG-Brd4.552 mice after 2 weeks of dox treatment (left and middle) and CAG^rtTA3/+^; TtG-Brd4.552 skin after 5 weeks of dox treatment and dox withdrawal (right).

Consistent with the expression pattern, Brd4 silencing induced hyperplasia in the epidermis and hair follicles, with orthokeratotic hyperkeratosis and moderate follicular keratosis (Figures 3A and S3C). Hair shafts were frequently deformed, including shafts that were wavy (zigzag), bent, giant, or attenuated, and hair follicles were more numerous, diverse in size, dilated, and often empty, with concomitant increases in the size of sebaceous glands. Brd4 suppression resulted in multiple layers of cytokeratin 6 (CK6)-positive cells along the inner root sheath, and we noted expanded E-cadherin expression upon Brd4 knockdown, indicative of hyperplasia and substantial defects in cellular architecture throughout the follicular and epidermal epithelium (Figures 3A and S3D). Importantly, the skin phenotype presented similarly in the two independent shBrd4 strains, suggesting a bona fide on-target effect of Brd4 suppression.

In our tet-regulated shRNA mice, Brd4 expression can be restored through removal of dox, allowing us to examine whether the observed skin effects are permanent or reversible. For this, TtG-Brd4 and littermate control mice were maintained on a dox diet until alopecia developed (5–7 weeks) and then returned to normal chow. Within 2 weeks following Brd4 restoration, hair growth was fully restored, hair shaft abnormalities disappeared, and histologic parameters of follicular and epidermal epithelia returned to normal (Figures 3A–3C and S3E). Thus, the skin phenotypes produced by sustained Brd4 suppression are rapidly reversible.

### Brd4 Silencing Depletes Secretory Cells and Lgr5^+^ Stem Cells in the Intestine

**Figure 4:**
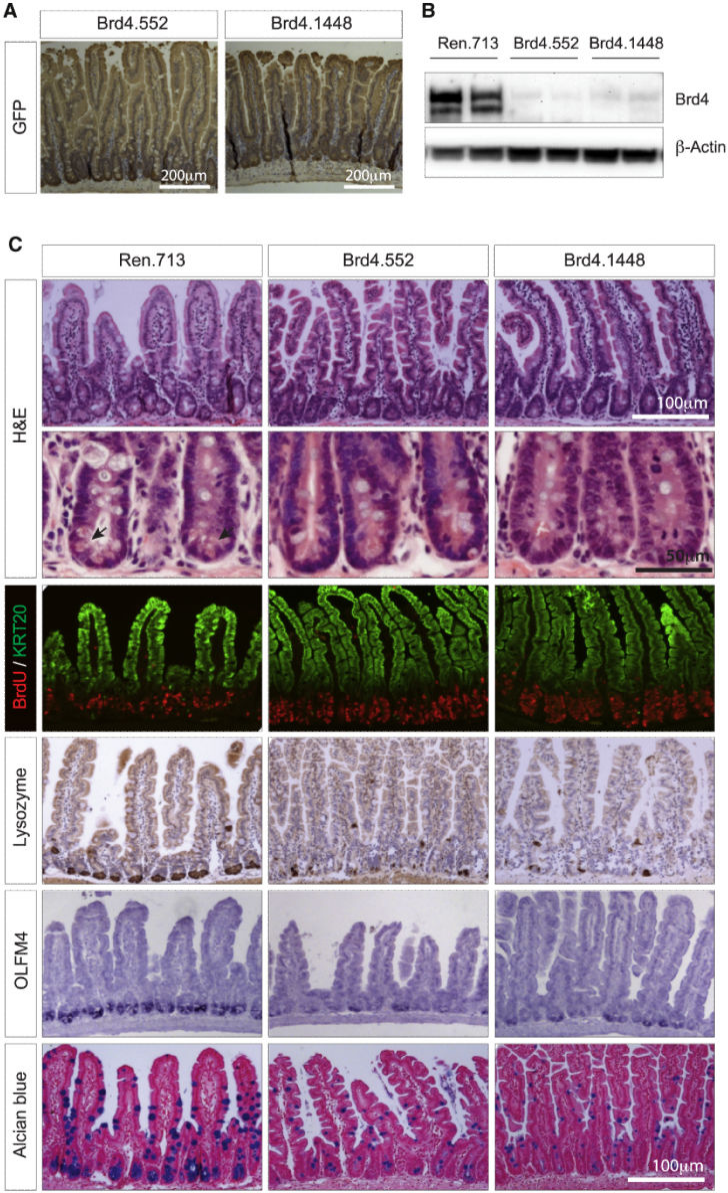
Brd4 Suppression Results in Loss of Intestinal Cellular Diversity (A) Immunohistochemical staining showing GFP expression (brown) in intestines of shBrd4 mice on the dox diet. Tissues were counterstained with hematoxylin. (B) Western blot of Brd4 protein levels in intestinal villi from CAG^rtTA3^-expressing TtG-Ren.713, TtG-Brd4.552, and TtG-Brd4.1448 mice, maintained on a dox diet for 2 weeks. (C) Histological and immunohistochemical stains of small intestine sections from CAG^rtTA3^-expressing TtG-Ren.713, TtG-Brd4.552, and TtG-Brd4.1448 mice. Included are H&E stains (where arrows indicate the location of eosinophilic granules of Paneth cells), immunofluorescent stains for BrdU incorporation (marking proliferating cells) and Keratin 20 (KRT20-differentiated cells), immunohistochemical staining for Lysozyme (Paneth cells), in situ hybridization for Olfm4 (intestinal stem cells), and Alcian blue stains (goblet cells). Scale bars are indicated.

Due to its high proliferative rate, the intestine is a common site of chemotherapy-associated toxicities. In CAG-rtTA3; TtG-Brd4 double-transgenic mice, the intestinal epithelium showed robust expression of GFP and suppression of Brd4 protein (Figures 4A, 4B, and S2C). In contrast to the dramatic hyperplastic response observed in the skin, the proliferation and differentiation of the intestine (indicated by bromodeoxyuridine [BrdU] incorporation and Keratin 20 staining) looked superficially normal (Figure 4C). Still, while moderate Brd4 silencing driven by R26-rtTA did not cause any disruptions to the intestinal composition (Figure S4A), strong Brd4 suppression with CAG-rtTA3 led to a marked depletion of eosinophilic-granule-containing Lysozyme^+^ Paneth cells at the base of the intestinal crypts, fewer and smaller Mucin^+^ goblet cells (Figure 4C), and a complete lack of Dclk1^+^ Tuft cells (Figure S4B). This clear difference implies that the level of Brd4 suppression required to induce intestinal phenotypes lies somewhere in between that produced by R26-rtTA and CAG-rtTA3 (Figure S2C).

In addition to the depletion of secretory lineage populations in CAG-rtTA3; TtG-Brd4 mice, Brd4 suppression caused a loss of Olfm4^+^/Lgr5^+^ stem cells at the base of intestinal crypts (Figures 4C and S4B). Disruption of the crypt and loss of Paneth cells was apparent within 8 days of dox treatment, sustainable for multiple weeks and presented similarly in both shBrd4 strains, implying it was a direct result of Brd4 suppression (Figure S4C). Notably, no changes in proliferation or apoptosis in the base of intestinal crypts were detected upon Brd4 depletion (Figures S4D and S4E), raising the possibility that Brd4 may control lineage specification in the gut rather than survival of specific cell types.

### Brd4 Knockdown Suppresses Organoid Formation

**Figure 5:**
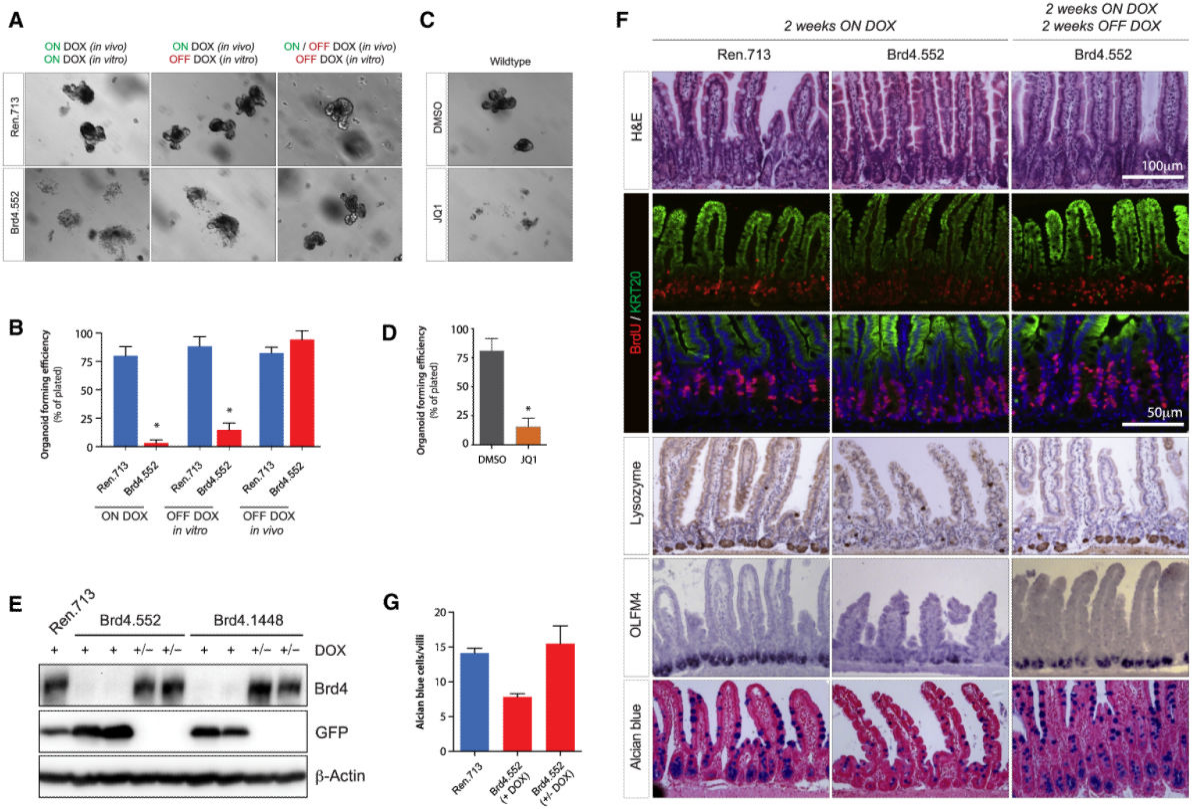
Brd4 Suppression by RNAi and JQ1 Inhibits Intestine Organoid Formation (A) Representative images of intestine crypt cultures from CAG^rtTA3^-expressing TtG-Ren.713 and TtG-Brd4.552 mice. Animals were fed a dox diet for 2 weeks and intestine crypts maintained on dox in vitro (left), crypts cultured off dox in vitro (center), or animals were fed dox-diet for 2 weeks, withdrawn for 2 weeks, and maintained off dox in vitro (right). (B) Quantification of organoid-forming efficiency for cultures shown in (A). (C) Representative images of C57Bl/6 intestine crypts when cultured in the presence of DMSO or 100 nM JQ1. (D) Quantification of organoid-forming efficiency for crypt cultures shown in (C). (E) Western blot of Brd4 protein levels in intestinal villi from CAG^rtTA3^-expressing TtG-Ren.713, TtG-Brd4.552, and TtG-Brd4.1448 mice, maintained on a dox diet for 2 weeks (+) or treated with dox for 2 weeks and withdrawn for 2 weeks (+/−). The expression of GFP and β-Actin are also shown. (F) H&E, BrdU, Keratin 20 (KRT20), lysozyme (Paneth cell), OLFM4 (stem cell), and Alcian blue (goblet cell) staining of small intestine in CAG^rtTA3^-expressing TtG-Ren.713 and TtG-Brd4.552 mice fed a dox diet for 2 weeks and in Brd4.552 mice following 2 weeks of dox withdrawal. Asterisks (*) indicate a statistically significant difference between shRen.713 and shBrd4.552 organoid-forming efficiency (p < 0.05), as determined by two-tailed Student’s t test. (G) Quantification of Alcian blue-positive goblet cells in CAG^rtTA3^-expressing TtG-Ren.713 and TtG-Brd4.552 mice after 2 weeks on dox and CAG^rtTA3/+^; TtG-Brd4.552 mice taken off dox for 2 weeks. Error bars represent the mean of three independent samples ± SEM. Data in (B) and (D) represent mean ± SD (n ≥ 3).

To determine whether the decrease in Olfm4 and Lgr5 staining in shBrd4 mice reflected a simple loss of gene expression or a loss of functional stem cells, we used a recently described intestinal crypt “organoid’ culture system (Sato et al., 2011). In this ex vivo culture system, organoid-forming ability is directly related to the functionality of Lgr5^+^ stem cells and niche-supporting Paneth cells (Durand et al., 2012, Sato et al., 2011). Consistent with a reduction in stem cell numbers, crypts isolated from shBrd4 mice on dox for 2 weeks could not form proliferative intestinal organoids compared to crypts from control mice (Figures 5A, 5B, S5A, and S5B). Impaired organoid formation was most apparent when crypts were maintained in the presence of dox ex vivo but was also significantly reduced when crypts from dox-treated mice were cultured in the absence of dox, suggesting that reduced colony-forming ability was largely established prior to crypt explant.

The substantial pathologies associated with Brd4 silencing are apparently at odds with the limited toxicities reported for BET inhibitors to date. Indeed, treatment of control C57Bl/6 mice with a pharmacological BET inhibitor (JQ1, once daily, 100 mg/kg intraperitoneally) for 2 weeks did not induce changes in the histological appearance of the small intestine or loss of Paneth cells (Figure S5C). Consistent with this, treatment of mice with JQ1 for 2 weeks had no detrimental effect on organoid-forming ability (not shown). It is not clear whether the different effects of shBrd4-mediated knockdown and JQ1 treatment on the intestine arise from intrinsic differences between Brd4 protein depletion and small-molecule BET inhibition or from poor drug stability/tissue delivery in vivo; JQ1 is known to have poor pharmacokinetic properties in vivo (Matzuk et al., 2012). However, ex vivo treatment of freshly isolated wild-type crypts with JQ1 (100 nM) strongly suppressed organoid formation (Figures 5C and 5D), similar to the effect of RNAi-mediated Brd4 suppression in vivo. Thus, both Brd4 suppression and small molecule BET protein inhibition (with JQ1) have similar effects on crypt health ex vivo, under conditions where drug delivery is not limiting.

To examine whether Brd4 silencing initiates a permanent change in stem cell function in the intestine, we treated TtG-Brd4 mice with dox for 2 weeks (when stem cell depletion is apparent), then removed dox to restore endogenous Brd4 expression (Figure 5E) and isolated intestinal crypts. Similar to what we observed in the skin, 2 weeks following dox withdrawal, crypts from shBrd4 mice appeared histologically normal, reacquired Paneth cells, goblet cells, and Olfm4^+^ stem cells (Figures 5F and 5G). Most importantly, the crypts from Brd4-restored animals contained functional stem cells that allowed efficient organoid formation in explant culture, indistinguishable from TtG-Ren.713 control mice (Figures 5A and 5B). Thus, while Brd4 silencing induces depletion of multiple cell types in the intestine, the effects are rapidly reversible, implying that the consequences of BET protein inhibition could be managed by appropriate dosing.

### Brd4 Suppression Sensitizes Mice to Radiation-Induced Intestinal Damage

**Figure 6:**
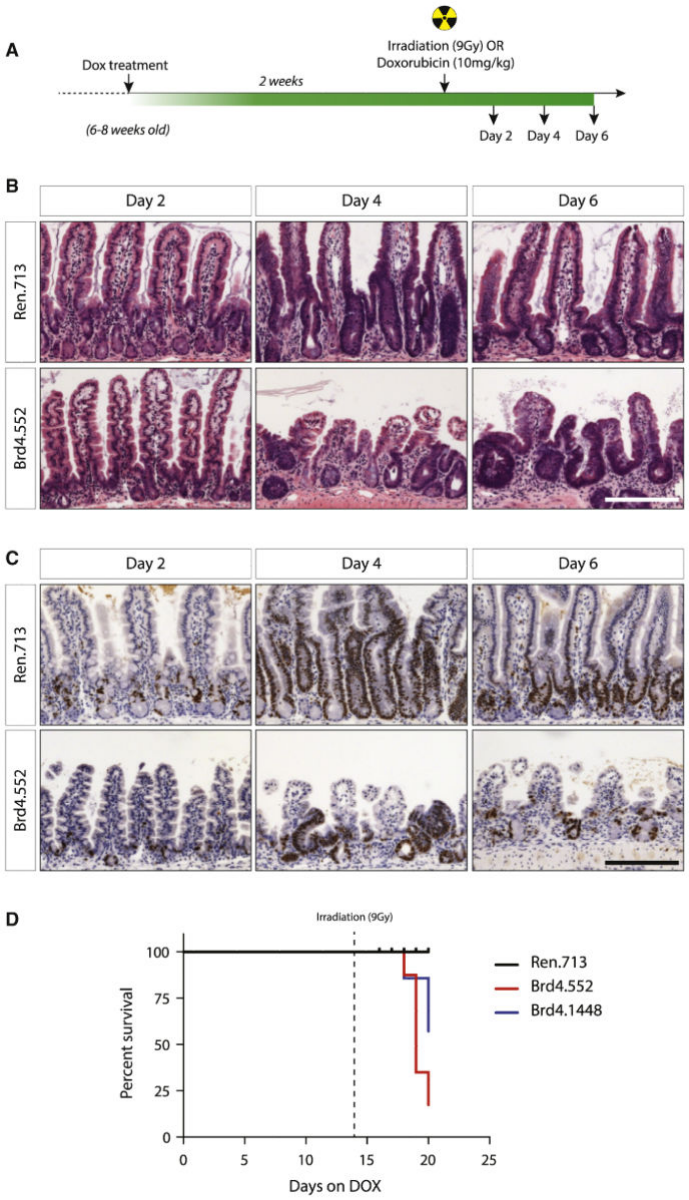
Brd4 Silencing Impairs Intestinal Regeneration following Irradiation (A) Schematic representation of the experimental timeline. Animals were treated with dox for 2 weeks and given a single, 9 Gy dose of γ-radiation. (B and C) H&E (B) and Ki67 (C) stains of small intestine sections from CAG^rtTA3^-expressing TtG-Ren.713 and TtG-Brd4.552 mice 2, 4, and 6 days following irradiation as indicated. Scale bars represent 100 μm. (D) Kaplan-Meier plot of dox-treated, irradiated mice, beginning at day 0 of dox treatment. Dotted line at day 14 indicates the day of irradiation.

The relative fitness of mice in which Brd4 was suppressed was surprising given the dramatic changes in intestinal cell composition. Nonetheless, such defects might reduce the ability of the intestine to recover in response to stress, for example, as might occur in response to cytotoxic therapies used to treat cancer. In fact, Lgr5^+^ crypt stem cells are critical for recovery from radiation-induced intestinal damage (Metcalfe et al., 2014). Similarly, the chemotherapeutic drug Adriamycin (doxorubicin hydrochloride), commonly used to treat AML, also induces intestinal damage, although in this case Lgr5^+^ stem cells are thought to play a less critical role in recovery (Dekaney et al., 2009). To determine whether shBrd4 mice were susceptible to intestine regenerative challenge posed by DNA-damaging-agent exposure, we treated control and TtG-Brd4 mice with dox for 2 weeks and induced acute DNA damage with either a single sublethal dose (9 Gy) of γ-radiation or a well-tolerated, single dose (10 mg/kg) of doxorubicin (Figure 6A).

Upon treatment with irradiation, we observed strikingly different outcomes in shBrd4 and control mice on dox. TtG-Ren.713 control animals showed transient weight loss associated with intestinal damage (Figure S6A) but stabilized 4 days following irradiation, accompanied by characteristic intestinal regeneration and expansion of the proliferative crypt compartment (Figures 6B and 6C). Six days following irradiation, the intestines of control animals looked normal. In stark contrast, Brd4-silenced intestines showed irregular, atrophied villi and inconsistent proliferative crypt recovery (Figures 6B, 6C, and S6B). Unlike control mice, Brd4-depleted animals continued to lose weight, owing to an inability to restore the absorptive epithelia, and most animals had to be sacrificed (Figures 6D and S6A). In combination with doxorubicin, Brd4-depleted mice demonstrated slightly impaired weight gain relative to control mice over the 7-day time period following the doxorubicin treatment (Figure S6C), suggestive of a mild defect in full recovery of intestinal function. However, histological examination of intestines did not indicate an effect on overall tissue integrity or differences in proliferative responses of control and shBrd4 groups at this dose of doxorubicin, possibly pointing to a more subtle defect (Figure S6D). Collectively, these data suggest that potent depletion of Brd4 impairs the ability of animals to fully respond to challenges that trigger intestinal regeneration, although the level of combined toxicity will likely depend on the type and dose of damaging agent.

## DISCUSSION

The BET family protein Brd4 is a promising therapeutic target for the treatment of cancer, and early-generation BET inhibitors are being evaluated in phase 1/2 clinical trials. Here, we used RNAi to explore the consequence of acute and potent Brd4 suppression in normal adult mouse tissues with the goal of predicting potential on-target toxicities associated with Brd4-targeted therapies in the clinic. More generally, the transgenic RNAi technology employed in our study enables us to spatially and reversibly modulate endogenous gene expression, a feature that allows us to explore an aspect of biology that cannot be easily achieved with traditional knockout mouse models. Consequently, this approach can be applied to explore the requirement for any potential drug target in normal tissue development and maintenance (thereby predicting side effects and susceptible tissues/organs of pharmacological inhibitors prior to clinical application).

In this study, the impact of potent Brd4 silencing in vivo was remarkably diverse. We observed dramatic and opposing effects on proliferation, differentiation, and the homeostasis of different tissues of the same animal. In the hematopoietic compartment, Brd4 silencing led to depletion of T lymphoid cells and Lineage^−^ Sca1^+^ cKit^+^ hematopoietic stem cells. In the skin, Brd4 knockdown caused significant architectural disruptions in the hair follicles that manifested in dramatic hair loss, while the epidermis showed hyperplasia, which was unexpected given the published role for Brd4 in normal cell-cycle progression (Dey et al., 2009; Yang et al., 2008). By contrast, in the intestine, we noted a substantial decrease in differentiation toward secretory cell lineages and a loss of functional stem cells. Though this stem cell depletion had limited effect under normal conditions, Brd4-suppressed intestines were hypersensitive to cytotoxic damage and could not properly initiate or sustain intestinal regeneration following irradiation. What mediates these diverse phenotypes in the skin and intestine is unclear, but it possibly occurs through mechanisms that are independent of the regulation of *Myc*, as has been reported in other non-hematopoietic contexts (Huang et al., 2009; Lockwood et al., 2012).

Using our transgenic shRNA mice, we focused our study on the intestine and skin, as these were two organs in which we could achieve strong, ubiquitous shRNA expression from the TREtight promoter and could therefore confidently examine tissue-autonomous effects of Brd4 suppression. Unfortunately, we were unable to express TREtight-driven shRNAs in a number of tissues (such as the liver) that we predict would be affected by strong Brd4 knockdown on the basis of the Brd4 heterozygote phenotype reported by Beddington and colleagues (Houzelstein et al., 2002). Thus, the consequences of systemic Brd4 depletion may be more numerous than reported here, and further work will be required to fully ascribe the role of Brd4 in all adult tissues.

Our study identified a range of potential on-target toxicities of Brd4 depletion that were not anticipated from preclinical studies using first-generation BET inhibitors such as JQ1. Specifically, in contrast to the apparent lack of impact of BET inhibitors on normal hematopoiesis, we observed depletion in T lymphocytes and stem cells. Furthermore, JQ1 treatment in our study did not result in loss of intestinal stem, Paneth, and secretory cells, which we consistently observed with our shRNA transgenic mice. While it remains possible that inherent differences between RNAi-mediated knockdown and small-molecule inhibition of Brd4 bromodomain function contribute to their differential impact on the intestine in vivo, it seems possible that this discrepancy can be explained by the poor pharmacokinetic properties of JQ1, which may preclude sustained target inhibition in vivo (Matzuk et al., 2012). Accordingly, previous work indicates RNAi and BETi induce similar gene expression changes (Zuber et al., 2011b), and we show here that the impact of Brd4 shRNAs and JQ1 on intestinal crypt function in vitro is similar. While we focused on normal tissues, it will be key to assess whether the level and duration of Brd4 disruption reported here to disrupt tissue homeostasis is required for meaningful therapeutic effects in tumors or whether an acceptable therapeutic index of BET inhibitors can be achieved in the clinic.

Perhaps most relevant to their eventual clinical use, our data suggest that potent Brd4 knockdown reduces the ability of animals to respond to at least some intestinal regenerative challenges that might be encountered during combinatorial therapeutic application of DNA-damaging agents. As shown here, the deleterious consequences of a particular combination will depend on the cytotoxic agent and likely the extent of BET protein inhibition. While the precise bases for these differences remain to be determined, the potential for synergistic toxicities for each combination should be examined carefully as combination trials are developed. Most importantly, however, the phenotypic consequences of Brd4 suppression appear completely reversible upon restoration of Brd4 expression, implying that any deleterious consequences of on-target Brd4 inhibition observed clinically could be managed through careful timing of drug administration and withdrawal.

## EXPERIMENTAL PROCEDURES

### Generation of ESC-Derived Mice

miR30-based shRNA targeting vectors were cloned as previously described (Dow et al., 2012) The shRNAs used in this study are listed as XhoI/EcoRI fragments in Table S1. Embryonic stem cells (ESCs) were targeted and screened as described previously (Dow et al., 2012; Premsrirut et al., 2011, 2013) and mice generated by tetraploid embryo complementation.

### Transgenic Animals and Ethics Approvals

All experimental procedures described in this study were approved by, and adhered to guidelines of, the Memorial Sloan-Kettering Cancer Center institutional animal care and use committee. Doxycycline was administered to mice via 625 mg/kg doxycycline-containing food pellets (Harlan Teklad).

### Nuclear Protein Extraction and Western Blotting

Cytoplasmic proteins from single-cell suspensions of thymi were extracted using hypotonic buffer and Triton X-100 followed by centrifugation. Nuclear proteins were then extracted through incubation with hypertonic buffer and quantified by DC protein assay (Bio-Rad). Intestinal villi were lysed in Laemmli buffer. Protein extracts were separated by SDS-PAGE and transferred onto polyvinylidene fluoride membrane (Millipore) for detection with antibodies.

### Immunohistochemistry and Immunofluorescence

Murine tissues were fixed overnight in 10% neutral buffered formalin or fresh 4% paraformaldehyde. Antigen retrieval was performed in Tris buffer for all immunohistochemical stains except for the detection of Lysozyme, where antigen retrieval was achieved by proteinase-K. ImmPRESS horseradish peroxidase-conjugated secondary antibodies together with ImmPact DAB (Vector labs) were used for chromagen development. Tissues were counterstained with hematoxylin. The general protocol for in situ hybridization for Olfm4 and Lgr5 were performed essentially as previously described (Gregorieff et al., 2005). For immunofluorescence studies, antigen retrieval was performed with citrate buffer. Slides were incubated overnight with primary antibodies and counterstained with DAPI.

### Intestine Crypt Isolation and Culture

Intestine crypt isolation and culture was done as previous described (Sato et al., 2011). For determination of organoid-forming efficiency, freshly isolated crypts were plated in triplicate and assessed 12 hr later to count viable crypts. The same microscopic fields were examined at day 4 and the data were normalized as relative crypt-forming efficiencies (proliferative organoids at day 4/viable crypts at 12 hr).

### Two-Color Competitive RNAi Assay and Hematopoietic Reconstitution

Two-color in vivo RNAi hematopoietic reconstitution assays were performed as described previously (Zuber et al., 2011a). Control (LMN-Cherry) and experimental (LMN-GFP) shRNA populations were mixed 1:1 and injected intravenously into recipient mice. Following hematopoietic reconstitution, the spleen, thymus, and bone marrow were analyzed for the presence of GFP^+^ and Cherry^+^ fluorescence markers in specific hematopoietic lineages by flow cytometry.

## Supplementary Material

Supplemental

## Highlights

Transgenic RNAi identifies potential toxicities associated with Brd4 inhibition

Brd4 suppression disrupts tissue homeostasis in multiple organs in adult mice

Brd4 silencing induces intestinal stem cell loss and hypersensitivity to irradiation

Deleterious phenotypes associated with Brd4 suppression are reversible

## In Brief

Pharmacological BET bromodomain inhibitors are entering early-phase clinical trials for the treatment of cancer. Here, Bolden et al. have used inducible and reversible transgenic RNAi to reveal on-target toxicities associated with strong Brd4 suppression. Their study provides insight into Brd4 function in normal adult tissues and predicts outcomes that are important for further development and clinical application of BET inhibitors.

## References

[R1] Belkina AC, Denis GV (2012). BET domain co-regulators in obesity, inflammation and cancer. Nat. Rev. Cancer.

[R2] Dekaney CM, Gulati AS, Garrison AP, Helmrath MA, Henning SJ (2009). Regeneration of intestinal stem/progenitor cells following doxorubicin treatment of mice. Am. J. Physiol. Gastrointest. Liver Physiol.

[R3] Delmore JE, Issa GC, Lemieux ME, Rahl PB, Shi J, Jacobs HM, Kastritis E, Gilpatrick T, Paranal RM, Qi J (2011). BET bromodomain inhibition as a therapeutic strategy to target c-Myc. Cell.

[R4] Dey A, Nishiyama A, Karpova T, McNally J, Ozato K (2009). Brd4 marks select genes on mitotic chromatin and directs postmitotic transcription. Mol. Biol. Cell.

[R5] Dow LE, Premsrirut PK, Zuber J, Fellmann C, McJunkin K, Miething C, Park Y, Dickins RA, Hannon GJ, Lowe SW (2012). A pipeline for the generation of shRNA transgenic mice. Nat. Protoc.

[R6] Durand A, Donahue B, Peignon G, Letourneur F, Cagnard N, Slomianny C, Perret C, Shroyer NF, Romagnolo B (2012). Functional intestinal stem cells after Paneth cell ablation induced by the loss of transcription factor Math1 (Atoh1). Proc. Natl. Acad. Sci. USA.

[R7] Filippakopoulos P, Qi J, Picaud S, Shen Y, Smith WB, Fedorov O, Morse EM, Keates T, Hickman TT, Felletar I (2010). Selective inhibition of BET bromodomains. Nature.

[R8] Floyd SR, Pacold ME, Huang Q, Clarke SM, Lam FC, Cannell IG, Bryson BD, Rameseder J, Lee MJ, Blake EJ (2013). The bromodomain protein Brd4 insulates chromatin from DNA damage signalling. Nature.

[R9] French CA, Miyoshi I, Aster JC, Kubonishi I, Kroll TG, Dal Cin P, Vargas SO, Perez-Atayde AR, Fletcher JA (2001). BRD4 bromodomain gene rearrangement in aggressive carcinoma with translocation t(15;19). Am. J. Pathol.

[R10] Grayson AR, Walsh EM, Cameron MJ, Godec J, Ashworth T, Ambrose JM, Aserlind AB, Wang H, Evan GI, Kluk MJ (2014). MYC, a downstream target of BRD-NUT, is necessary and sufficient for the blockade of differentiation in NUT midline carcinoma. Oncogene.

[R11] Gregorieff A, Pinto D, Begthel H, Destrée O, Kielman M, Clevers H (2005). Expression pattern of Wnt signaling components in the adult intestine. Gastroenterology.

[R12] Houzelstein D, Bullock SL, Lynch DE, Grigorieva EF, Wilson VA, Beddington RS (2002). Growth and early postimplantation defects in mice deficient for the bromodomain-containing protein Brd4. Mol. Cell. Biol.

[R13] Huang B, Yang XD, Zhou MM, Ozato K, Chen LF (2009). Brd4 coactivates transcriptional activation of NF-kappaB via specific binding to acetylated RelA. Mol. Cell. Biol.

[R14] Lockwood WW, Zejnullahu K, Bradner JE, Varmus H (2012). Sensitivity of human lung adenocarcinoma cell lines to targeted inhibition of BET epigenetic signaling proteins. Proc. Natl. Acad. Sci. USA.

[R15] Matzuk MM, McKeown MR, Filippakopoulos P, Li Q, Ma L, Agno JE, Lemieux ME, Picaud S, Yu RN, Qi J (2012). Small-molecule inhibition of BRDT for male contraception. Cell.

[R16] McJunkin K, Mazurek A, Premsrirut PK, Zuber J, Dow LE, Simon J, Stillman B, Lowe SW (2011). Reversible suppression of an essential gene in adult mice using transgenic RNA interference. Proc. Natl. Acad. Sci. USA.

[R17] Mertz JA, Conery AR, Bryant BM, Sandy P, Balasubramanian S, Mele DA, Bergeron L, Sims RJ (2011). Targeting MYC dependence in cancer by inhibiting BET bromodomains. Proc. Natl. Acad. Sci. USA.

[R18] Metcalfe C, Kljavin NM, Ybarra R, de Sauvage FJ (2014). Lgr5+ stem cells are indispensable for radiation-induced intestinal regeneration. Cell Stem Cell.

[R19] Mirguet O, Gosmini R, Toum J, Clément CA, Barnathan M, Brusq JM, Mordaunt JE, Grimes RM, Crowe M, Pineau O (2013). Discovery of epigenetic regulator I-BET762: lead optimization to afford a clinical candidate inhibitor of the BET bromodomains. J. Med. Chem.

[R20] Nicodeme E, Jeffrey KL, Schaefer U, Beinke S, Dewell S, Chung CW, Chandwani R, Marazzi I, Wilson P, Coste H (2010). Suppression of inflammation by a synthetic histone mimic. Nature.

[R21] Premsrirut PK, Dow LE, Kim SY, Camiolo M, Malone CD, Miething C, Scuoppo C, Zuber J, Dickins RA, Kogan SC (2011). A rapid and scalable system for studying gene function in mice using conditional RNA interference. Cell.

[R22] Premsrirut PK, Dow LE, Park Y, Hannon GJ, Lowe SW (2013). Creating transgenic shRNA mice by recombinase-mediated cassette exchange. Cold Spring Harb Protoc.

[R23] Sato T, van Es JH, Snippert HJ, Stange DE, Vries RG, van den Born M, Barker N, Shroyer NF, van de Wetering M, Clevers H (2011). Paneth cells constitute the niche for Lgr5 stem cells in intestinal crypts. Nature.

[R24] Wang R, Li Q, Helfer CM, Jiao J, You J (2012). Bromodomain protein Brd4 associated with acetylated chromatin is important for maintenance of higher-order chromatin structure. J. Biol. Chem.

[R25] Wu SY, Chiang CM (2007). The double bromodomain-containing chromatin adaptor Brd4 and transcriptional regulation. J. Biol. Chem.

[R26] Yang Z, He N, Zhou Q (2008). Brd4 recruits P-TEFb to chromosomes at late mitosis to promote G1 gene expression and cell cycle progression. Mol. Cell. Biol.

[R27] Zuber J, Rappaport AR, Luo W, Wang E, Chen C, Vaseva AV, Shi J, Weissmueller S, Fellmann C, Taylor MJ (2011a). An integrated approach to dissecting oncogene addiction implicates a Myb-coordinated self-renewal program as essential for leukemia maintenance. Genes Dev.

[R28] Zuber J, Shi J, Wang E, Rappaport AR, Herrmann H, Sison EA, Magoon D, Qi J, Blatt K, Wunderlich M (2011b). RNAi screen identifies Brd4 as a therapeutic target in acute myeloid leukaemia. Nature.

