# Supplementary material for: Inducible In Vivo Silencing of Brd4 Identifies Potential Toxicities of Sustained BET Protein Inhibition
Source: Cell Rep. Author manuscript; Available in PMC 2014 Nov 17. (PMC4234106; doi:10.1016/j.celrep.2014.08.025)
Supplement: Supplemental [file NIHMS640955-supplement-Supplemental.pdf]

Cell Reports, Volume *8*

Supplemental Information

Inducible In Vivo Silencing of Brd4 Identifies Potential Toxicities of Sustained BET Protein Inhibition

Jessica E. Bolden, Nilgun Tasdemir, Lukas E. Dow, Johan H. van Es, John E. Wilkinson, Zhen Zhao, Hans Clevers, and Scott W. Lowe

Inventory of Supplemental Information

1. Supplemental Experimental Procedures
2. Supplemental Figure Legends
3. Figures S1-S6
4. Table S1

Supplemental Experimental Procedures

MEF experiments

MEFs were isolated from E12.5 embryos and cultured in high glucose DMEM supplemented with 10% fetal bovine serum, 100U/mL penicillin and 100μg/mL streptomycin and 2mM L-glutamine. The expression of shRNAs was induced by addition of 1mg/mL doxycycline hyclate (Sigma) to the growth medium. MEFs were collected for western blotting by scraping into ice-cold PBS and pelleting at 400g for 5 minutes, prior to storage or lysis.

Nuclear protein extraction and western blotting

Thymi from transgenic mice were mechanically disrupted and passed through a 100μm filter to generate single cell suspensions. Cell pellets were stored at -80°C until lysis. Nuclear proteins extracted as follows: cells were swelled in hypotonic buffer [10mM Tris HCl pH 8.0, 1.5mM $MgCl_2$, 10mM KCl, 1x complete protease inhibitor cocktail (Roche)], and lysed by the addition of Triton X-100 to a final concentration of 0.25% (v/v). Extracts were immediately centrifuged at 2000g for 10 minutes and cytoplasmic fractions (supernatant) retained. Nuclear proteins were washed once with hypotonic buffer, pelleted at 2000g for 10 minutes, and extracted through incubation with hypertonic buffer (5mM Tris HCl pH 8.0, 1.5mM $MgCl_2$, 0.2mM EDTA, 0.5M NaCl, 25% (v/v) Glycerol, 1x complete protease inhibitor), for 1 hour on ice. Cellular debris was removed by centrifugation at 2000g for 10 minutes and nuclear proteins quantified by DC protein assay (Bio-Rad). Intestinal villi were lysed in Laemmli buffer. Protein extracts were separated by SDS-PAGE and transferred onto immobilon-P PVDF membrane (Millipore) and proteins detected with anti-Brd4 antibodies (Bethyl Labs A301-985A100 and Santa Cruz Biotechnology H-250), an anti-c-Myc antibody **(**Abcam, ab32072**)** and anti-β-actin (Sigma, A3854). Loading of nuclear extracts was determined by Ponceau S staining of PVDF membranes.

Immunohistochemistry and immunofluorescence

Murine tissues were fixed overnight in 10% neutral buffered formalin or fresh 4% paraformaldehyde, and transferred to 70% ethanol prior to paraffin embedding. Antigen retrieval was performed at high temperature and pressure for 5 minutes in 10mM Tris, 1mM EDTA (pH 9.0), 0.05% Tween-20 for all immunohistochemical stains except for the detection of Lysozyme, where antigen retrieval was achieved by proteinase-K (200mg/mL) digestion for 5 mins at RT. The following antibodies were used: anti-GFP (1:200, D5.1, Cell Signaling), anti-Ki67 (1:200, Sp6 clone, Abcam), anti-Dclk1 (Abcam, #ab37994) and anti-lysozyme (1:400 DAKO EC.3.2.1.17). ImmPRESS HRP-conjugated secondary antibodies together with ImmPact DAB (Vector labs) were used for chromagen development. Tissues were counterstained with hematoxylin. The general protocol for in situ hybridisation for Olfm4 and Lgr5 were performed

essentially as previously described (Gregorieff et al 2005). For immunofluorescence studies, antigen retrieval was performed at high pressure for 5 minutes in 10mM sodium citrate buffer pH 6.0 containing 0.05% tween-20, blocked with mouse-on-mouse block (Vector labs) for 1 hour, washed twice with wash buffer (PBS, 0.5% Triton X-100), blocked with 2.5% normal goat serum (in wash buffer) and incubated overnight with primary antibodies diluted in wash buffer at 4°C. Slides were washed three times prior to incubation with Alexa-fluor conjugated secondary antibodies for 1 hour at room temperature. Slides were washed three times, nuclei counterstained with DAPI (1μg/mL) and mounted with ProLong Gold mounting medium (Life Technologies). The following antibodies were used: anti-Brd4 (Bethyl Labs, A301-985A100, 1:100), anti-E-cadherin (Transduction labs C20820, 1:250), anti-GFP (Abcam Ab13970, 1:500), Alexa-fluor- anti-mouse-IgG, anti-rabbit-IgG and anti-chicken IgG (Life technologies, 1:1000).

H&E and immunostained tissue sections were imaged on a Zeiss Axio Imager Z.2 using 10x (NA 0.3), 20x (NA 0.5) objectives, coupled with a Hamamatsu CCD camera.

Intestinal damage

For intestinal challenge experiments, shBrd4 and shRen mice were treated with doxycycline for 2 weeks, then either treated with a single dose of 9Gy ionizing radiation, or given a single dose of Adriamycin (doxorubicin hydrochloride) 10mg/kg by intraperitoneal injection.

JQ1 preparation and administration

JQ1 powder was dissolved in DMSO to generate a concentrated 50mg/mL stock solution. For administration to animals, a working solution was generated by diluting 1 part of the concentrated JQ1 stock drop-wise into 9 parts 10% Hydroxypropyl beta cyclodextrin (Sigma C0926). C57Bl/6 mice received once daily intraperitoneal injections of 100mg/kg JQ1 for 2 weeks. *Ex-vivo*, JQ1 was added to crypt growth medium to a final concentration of 100nM. Doxycycline was added to crypt growth medium to a final concentration of 0.5μg/mL.

Intestine crypt isolation and culture:

Under sterile conditions, the anterior 15cm of mouse small intestine was isolated, opened longitudinally and washed extensively in ice-cold phosphate buffered saline (PBS) to remove intestinal contents. ~5mm lengths of 'clean' intestine were cut, placed in ice-cold sterile-filtered PBS/5mM EDTA, and villi released by vigorous pipetting. Villi-containing supernatant was discarded. Intestine pieces were first incubated with PBS/5mM EDTA on ice for 10 minutes, and again for 30 minutes, with villi released by inversion at the end of each EDTA/PBS incubation. Intestine pieces were washed once with PBS, and crypts released

into 10mL PBS by vigorous pipetting. 10mL crypt isolation medium [DMEM/F12 Advanced (Life Technologies), 100U/mL penicillin, 100μg/mL streptomycin (Life Technologies), 1x Glutamax (Life Technologies)] was added to released crypts, and passed through 100μm filter. The crypt-containing solution was next passed through a 70μm filter into a tube pre-coated with sterile 10% (w/v) bovine-serum albumin. Crypts were centrifuged at 150g for 5 minutes and resuspended in crypt growth media [DMEM/F12 Advanced, 100U/mL penicillin, 100μg/mL streptomycin, 1x glutamax, 40ng/mL murine recombinant epidermal growth factor (Peprotech #315-09), 50ng/mL murine recombinant Noggin (Peprotech #250-38) and 250ng/mL R-spondin (R&D systems, 3475-RS)]. Crypts were suspended in Matrigel basic membrane matrix (BD #354234), covered with crypt growth medium and incubated at 37°C, 5% CO_2. For determination of organoid forming efficiency, freshly isolated crypts were plated in triplicate and assessed 12 hours later to count viable crypts. The same microscopic fields were examined at day 4 and the data were normalized as relative crypt forming efficiencies (proliferative organoids at day 4 / viable crypts at 12hrs).

Two-color competitive RNAi assay and hematopoietic reconstitution

Two-color *in-vivo* RNAi hematopoietic reconstitution assays were performed as described (Zuber et al 2011a). The LMN retroviral vectors (MSCV-miR30-PGK-NeoR-IRES-GFP and MSCV-miR30-PGK-NeoR-IRES-Cherry) have been described elsewhere (Zuber et al 2011a), and retroviral supernatants were generated using established protocols with pCMV-gag/pol and pCMV-Eco-Env plasmids to aid viral packaging. Hematopoietic stem and progenitor cells (HSPCs) were isolated from E13.5-15 wild type c57Bl/6 (CD45.2$^+$) embryos, pooled and retrovirally transduced with LMN-based shRNAs against Brd4 (shBrd4.1448, shBrd4.552), Renilla luciferase (shRen.713 – neutral control) and Rpa3 (shRpa3.455 – positive control for strong depletion). 6-8 week old B6.SJL (CD45.1$^+$) recipient mice had been lethally irradiated with split doses of 5.5Gy and 5.0Gy, administered 24 hours and 1 hour prior to injection of transduced cells, respectively. Control (LMN-Cherry) and experimental (LMN-GFP) shRNA populations were mixed 1:1, and injected intravenously into recipient mice. Recipient mice were maintained on ciprofloxacin-containing drinking water for two weeks. Following hematopoietic reconstitution, the spleen, thymus and bone marrow were analyzed for the presence of GFP$^+$ and Cherry$^+$ fluorescence markers in specific hematopoietic lineages by flow cytometry with an LSRII flow cytometer (BD Biosciences) using the following antibodies: CD45.2 (1:100, Bio Legend, 109820), CD3 (1:300, Bio Legend, 100309), CD4 (1:100, Bio Legend, 100409), CD8 (1:500, Bio Legend, 100711), B220 (1:500, Bio Legend, 103211), Ter119 (1:500, Bio Legend, 116209), Gr1 (1:1000, Bio Legend, 108410) . FlowJo software (Tree Star) was used for flow cytometry analyses. Statistical analyses (unpaired two-tailed T-tests) were performed with GraphPad Prism (GraphPad Software Inc.).

Supplemental Figure Legends

Figure S1, related to Figure 1: Brd4 knockdown causes T cell depletion

(A) Immunoblot of Brd4 protein levels in fetal-liver derived hematopoietic stem cells, following transduction with a retroviral vector constitutively expressing Ren.713, Brd4.552 or Brd4.1448. (B) Competitive hematopoietic reconstitution in the spleen. The percentage of CD45.2^{+} cells expressing shRen.713 (red) and the indicated experimental hairpin (green), in specific hematopoietic lineages (B220+ B cells, CD3+ T cells and CD4/CD8 T cell subsets, Ter119+ erythroid cells and Gr1+ granulocytes) are shown. Data is presented as mean + SEM (N=4). Asterisks (*) indicate a statistically significant difference between the presence of neutral control and experimental shRNAs ($p<0.05$), as determined by a two-tailed Students T-test.

Figure S2, related to Figure 2: Characterization of transgenic shRNA mice

(A) Immunoblot of Brd4 and c-Myc in nuclear extracts from thymocyte preparations. 4 week old mice were fed a dox-containing diet for 2 weeks prior to collection and preparation of tissues for western analysis. Ponceau S indicates protein loading. (B) Bright field images and GFP expression in diverse tissues from TtG-Ren.713 mice when driven by R26-rtTA and CAG-rtTA3. (S) spleen (K) kidney (P) pancreas (SG) salivary gland (T) thymus. Mice were fed a dox diet for 2 weeks prior to tissue collection and images acquired using a GFP fluorescence stereoscope. (C) Immunoblot of Brd4 protein levels in intestinal villi from TtG-Ren.713, TtG-Brd4.552 and TtG-Brd4.1448 mice, when driven by either R26-rtTA or CAG-rtTA3. Mice were maintained on a dox diet for 2 weeks.

Figure S3, related to Figure 3: Brd4 suppression causes alopecia and hair follicle defects

(A) Weight changes (g) of male and female CAGs-rtTA3; TtG-Brd4.1448 mice on dox diet, relative to Day 0 of dox treatment. Littermate controls include double transgenic CAG$^{rtTA3/+}$; TtG-Ren.713 mice, and single transgenic mice that carry a TtG-shRNA but lack a tet-transactivator (B) Representative images of CAG $^{rtTA3/+}$; TtG-shBrd4.1448 and littermate control mice after 7 weeks on dox diet, and 5 weeks following dox withdrawal. (C) H&E stains of dorsal skin sections from CAG^{rtTA3}; TtG-Brd4.1448 following 7 weeks of dox treatment and 5 weeks of dox withdrawal (D) Immunofluorescent stains for Ki67 (with DAPI counterstain), cytokeratin 6 (CK6) and E-cadherin (E-Cad) in dorsal skin sections from CAG^{rtTA3}; TtGBrd4.1448 mice after 7 weeks of dox treatment. (E) Immunofluorescent stains for Brd4, E-Cadherin (E-Cad) in CAG^{rtTA3}; TtGBrd4.1448 dorsal skin after 7 weeks of dox treatment and 5 weeks of dox withdrawal.

Figure S4, related to Figure 4: Brd4 suppression causes loss of intestine stem cells and Tuft cells without apoptosis or loss of proliferation

(A) Immunohistochemical staining for the presence of Lysozyme (Paneth cells) in small intestines from R26-rtTA-expressing TtG-Ren.713, TtG-Brd4.552 and TtG-Brd4.1448 mice. Mice were fed a dox diet for 2 weeks (B) Stains of small intestine sections from $CAG^{rtTA3/+}$- expressing TtG-Ren.713, TtG-Brd4.552 and TtG-Brd4.1448 mice are shown. *In situ* hybridization analyses reveal reduced Lgr5 staining in the base of intestinal crypts in shBrd4 mice. Immunohistochemical stains show the absence of Dclk1 positive tuft cells from shBrd4 villi (C-E) Acute time course to examine intestine apoptosis and proliferation upon Brd4 knockdown by immunohistochemistry (C) loss of paneth cells (Lysozyme) (D) the presence of apoptotic cells (cleaved caspase 3) and (E) proliferation (Ki67) in the intestines of CAG^{rtTA3}- expressing TtG-Ren.713, TtG-Brd4.552 and TtG-Brd4.1448 mice. Mice were untreated, or fed a dox diet for 2, 4 or 8 days.

Figure S5, related to Figure 5: Suppression of Brd4 impairs intestine organoid forming efficiency

(A) Representative images of intestine crypt cultures from CAG^{rtTA3}-expressing TtG-Ren.713 and TtG-Brd4.1448 mice fed a dox-containing diet for 2 weeks, and maintained in the presence of dox *in vitro*. (B) Quantification of organoid forming efficiency where shRNA mice were fed a dox diet for 2 weeks, crypts isolated and then cultured either on dox, or off dox *in vitro*. Data represents the mean +/- SD ($n \geq 3$), Asterisks indicated $p<0.05$, two-tailed Students t-test. (C) Intestinal sections of C57Bl/6 mice administered JQ1 (once daily, 100mg/kg, i.p.) or vehicle alone for 2 weeks. Representative H&E images of small intestine sections are shown. Sections were immunohistochemically stained for the Paneth cell marker Lysozyme.

Figure S6, related to Figure 6: Brd4 silencing impairs recovery following irradiation

(A) Weight changes following irradiation of CAG^{rtTA3}-expressing TtG-Ren.713, TtG-Brd4.552 and TtG-Brd4.1448 mice treated with dox for 2 weeks. Body weight is plotted relative to starting weight on the day of irradiation. The Dox diet was maintained after irradiation. Error bars represent the mean +/- SEM, $n \geq 3$ for each time point, except Day 6 for Brd4.552 (n=2) as most animals had to be sacrificed by this time. (B) H&E and Ki67 stains of small intestine sections from $CAG^{rtTA3/+}$; TtG-Brd4.1448 mice 2, 4 and 6 days following irradiation as indicated. Scale bars represent 100μm. (C) Weight changes following doxorubicin treatment (single injection on D0, 10mg/kg, i.p) of CAG^{rtTA3}-expressing TtG-Ren.713, TtG-Brd4.552 and TtG-Brd4.1448 mice treated with dox for 2 weeks. Dox diet was maintained after doxorubicin treatment. Body weight is plotted relative to starting weight on the day of doxorubicin

administration. Data is presented as mean +/- SEM (n=3). (D) Ki67 stains of small intestine sections from CAG^{rtTA3}-expressing TtG-Ren.713 and TtG-Brd4.552 mice 2, 4 and 7 days following doxorubicin administration.

Figure S1

A

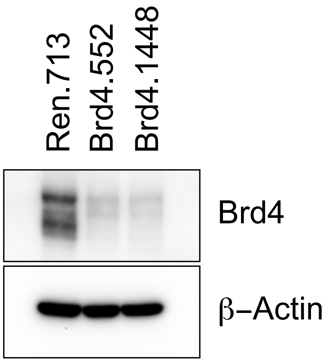
Ren.713
Brd4.552
Brd4.1448
Brd4
β–Actin

B

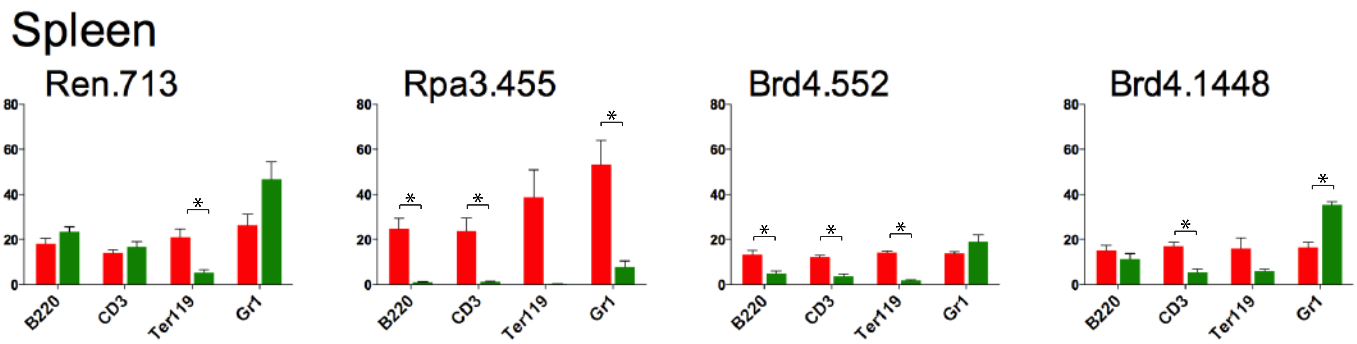
Spleen
Ren.713
Rpa3.455
Brd4.552
Brd4.1448
B220
CD3
Ter119
Gr1

Figure S2

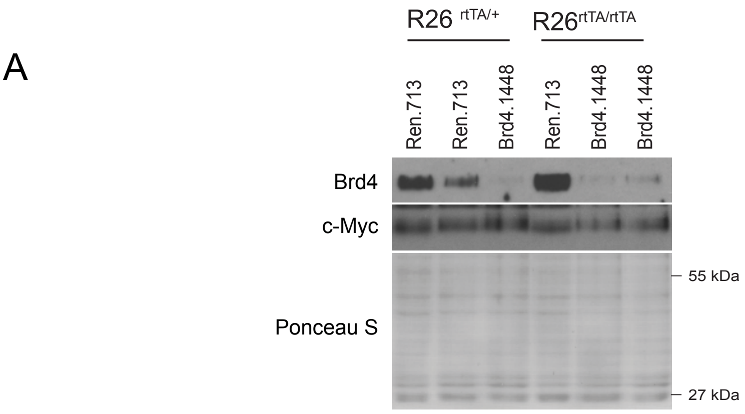
A
R26 rtTA/+
R26rtTA/rtTA
Ren.713
Ren.713
Brd4.1448
Ren.713
Brd4.1448
Brd4.1448
Brd4
c-Myc
Ponceau S
55 kDa
27 kDa

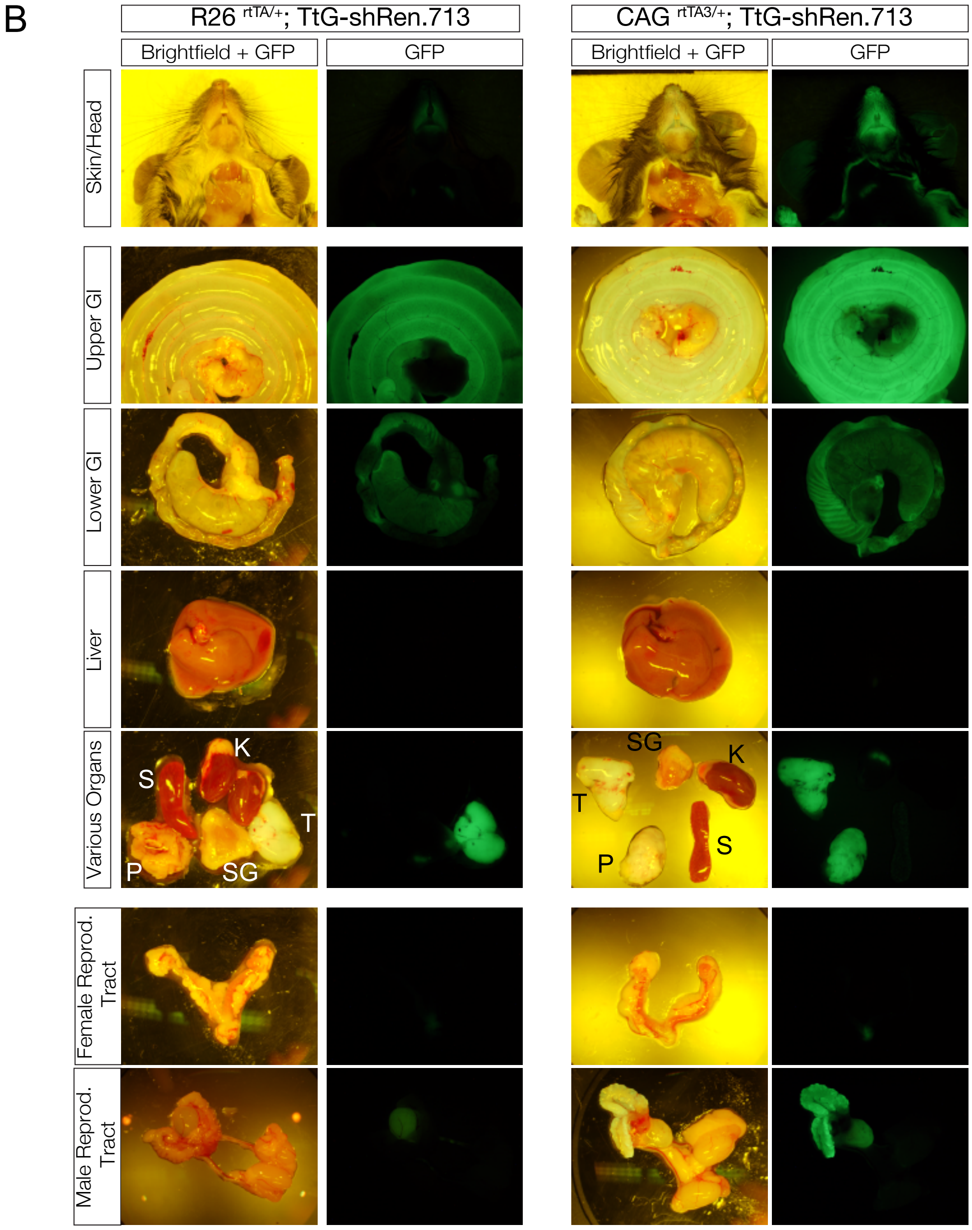
B
R26 rtTA/+; TtG-shRen.713
CAG rtTA3/+; TtG-shRen.713
Brightfield + GFP
GFP
Brightfield + GFP
GFP
Skin/Head
Upper GI
Lower GI
Liver
Various Organs
Female Reprod. Tract
Male Reprod. Tract
S
K
T
P
SG
SG
K
T
S
P

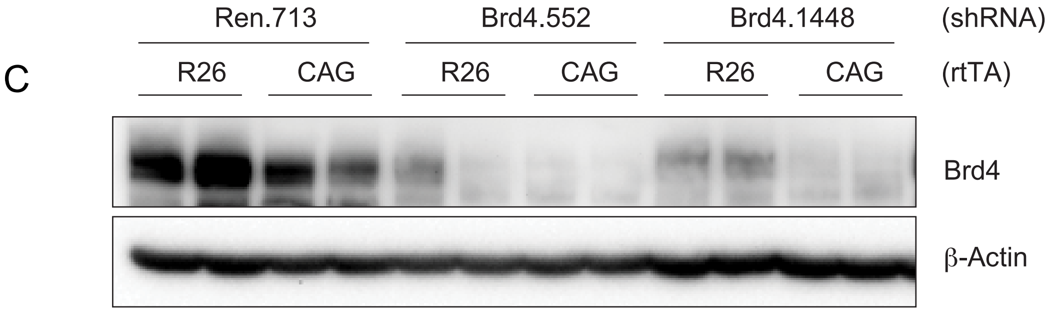
C
Ren.713
Brd4.552
Brd4.1448
(shRNA)
R26
CAG
R26
CAG
R26
CAG
(rtTA)
Brd4
β-Actin

Figure S3

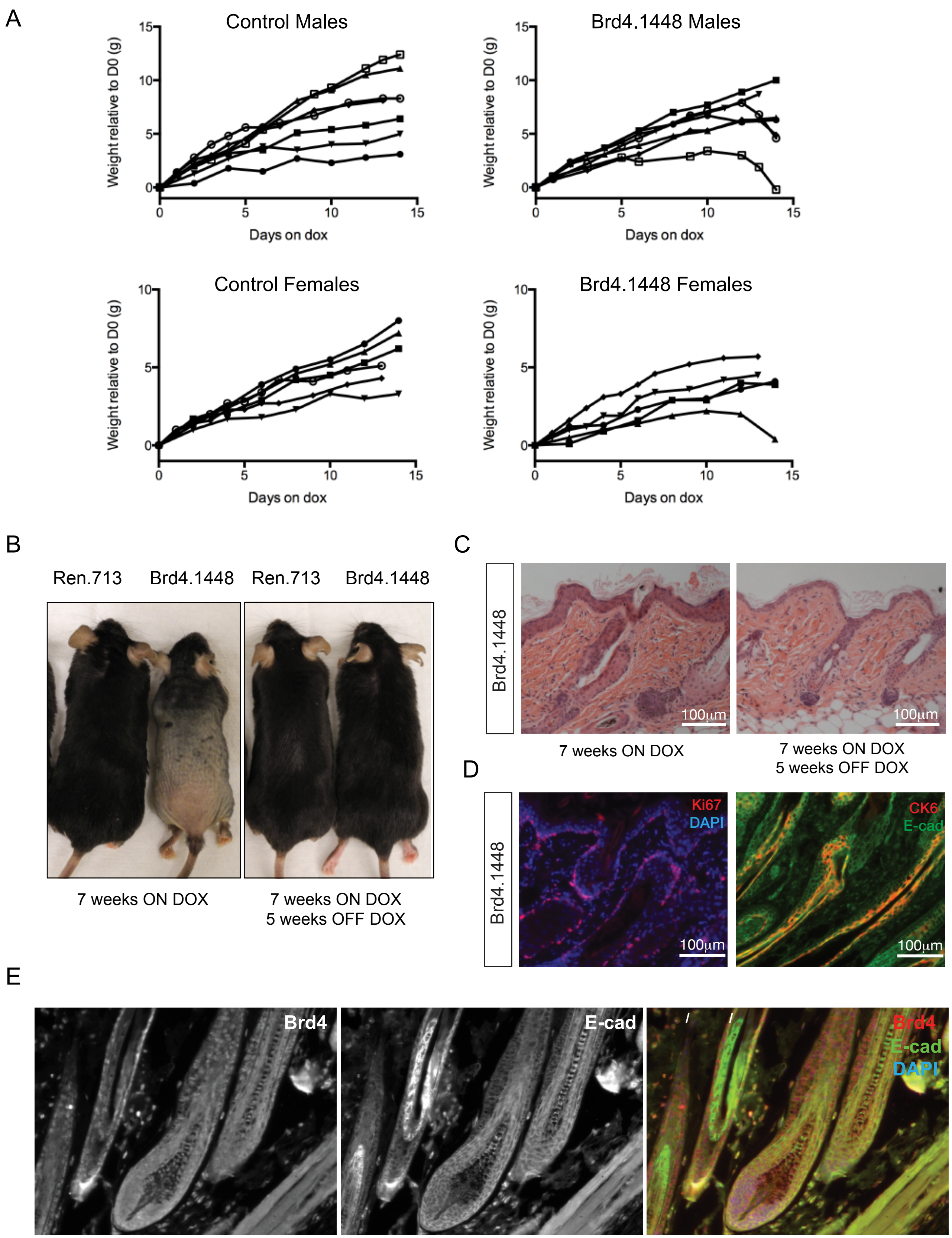
A
Control Males
Brd4.1448 Males
Control Females
Brd4.1448 Females
Weight relative to D0 (g)
Days on dox
B
Ren.713
Brd4.1448
Ren.713
Brd4.1448
7 weeks ON DOX
7 weeks ON DOX
5 weeks OFF DOX
C
Brd4.1448
100μm
100μm
7 weeks ON DOX
7 weeks ON DOX
5 weeks OFF DOX
D
Brd4.1448
Ki67
DAPI
CK6
E-cad
100μm
100μm
E
Brd4
E-cad
Brd4
E-cad
DAPI

Figure S4

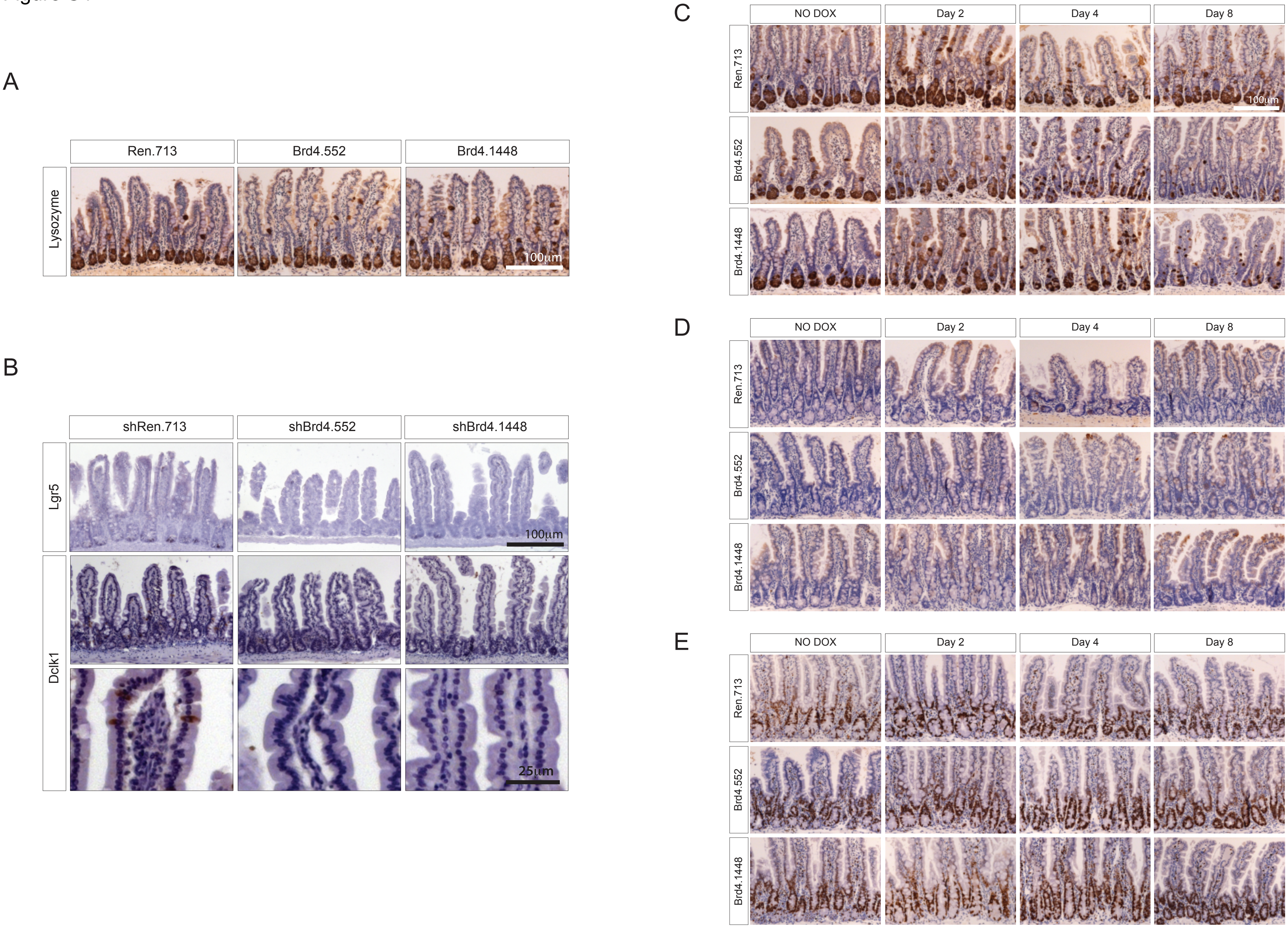
A
Ren.713
Brd4.552
Brd4.1448
Lysozyme
100μm
B
shRen.713
shBrd4.552
shBrd4.1448
Lgr5
100μm
Dclk1
25μm
C
NO DOX
Day 2
Day 4
Day 8
Ren.713
Brd4.552
Brd4.1448
100μm
D
NO DOX
Day 2
Day 4
Day 8
Ren.713
Brd4.552
Brd4.1448
E
NO DOX
Day 2
Day 4
Day 8
Ren.713
Brd4.552
Brd4.1448

Figure S5

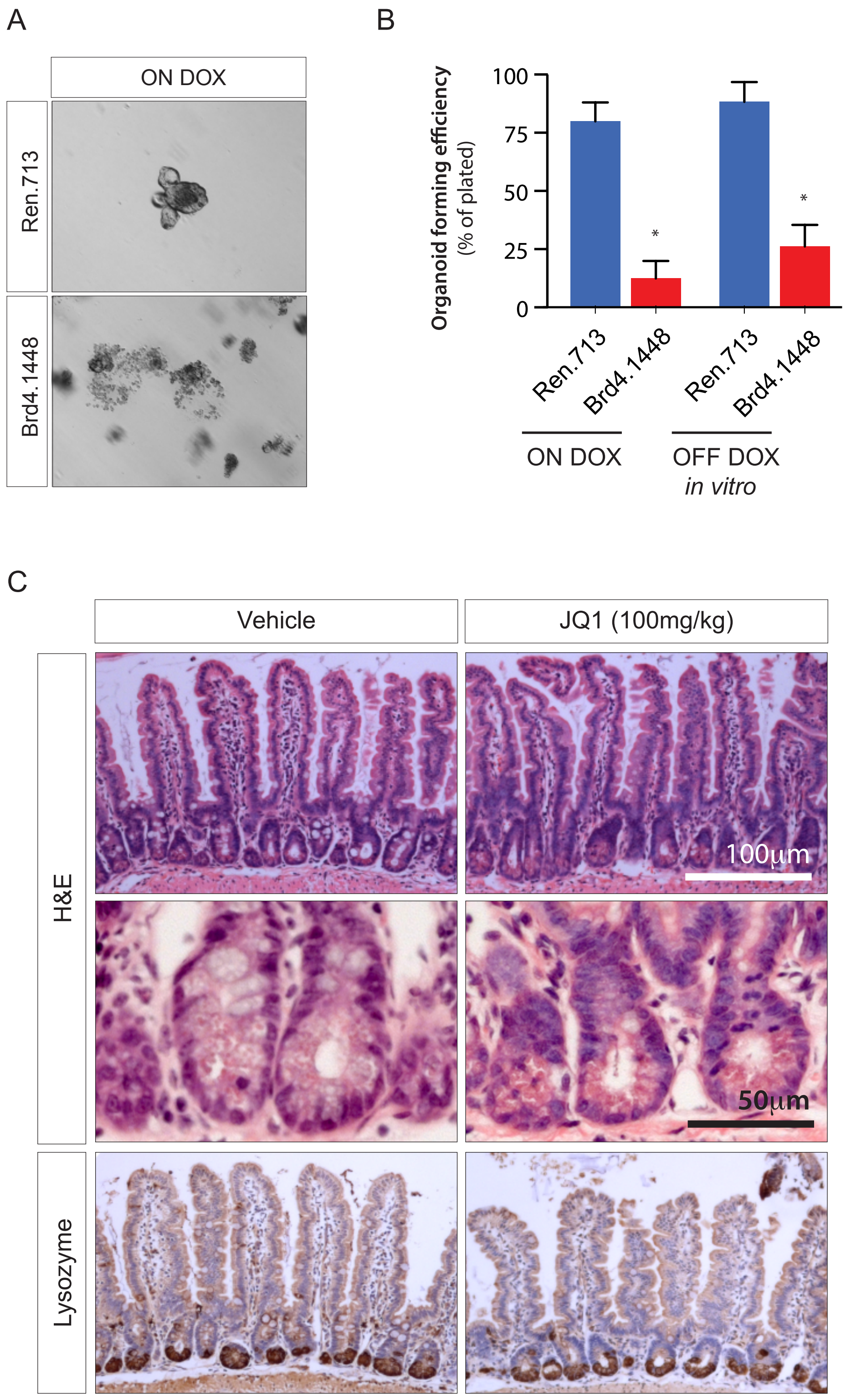
A
ON DOX
Ren.713
Brd4.1448
B
Organoid forming efficiency
(% of plated)
100
75
50
25
0
*
*
Ren.713
Brd4.1448
Ren.713
Brd4.1448
ON DOX
OFF DOX
in vitro
C
Vehicle
JQ1 (100mg/kg)
H&E
100μm
50μm
Lysozyme

Figure S6

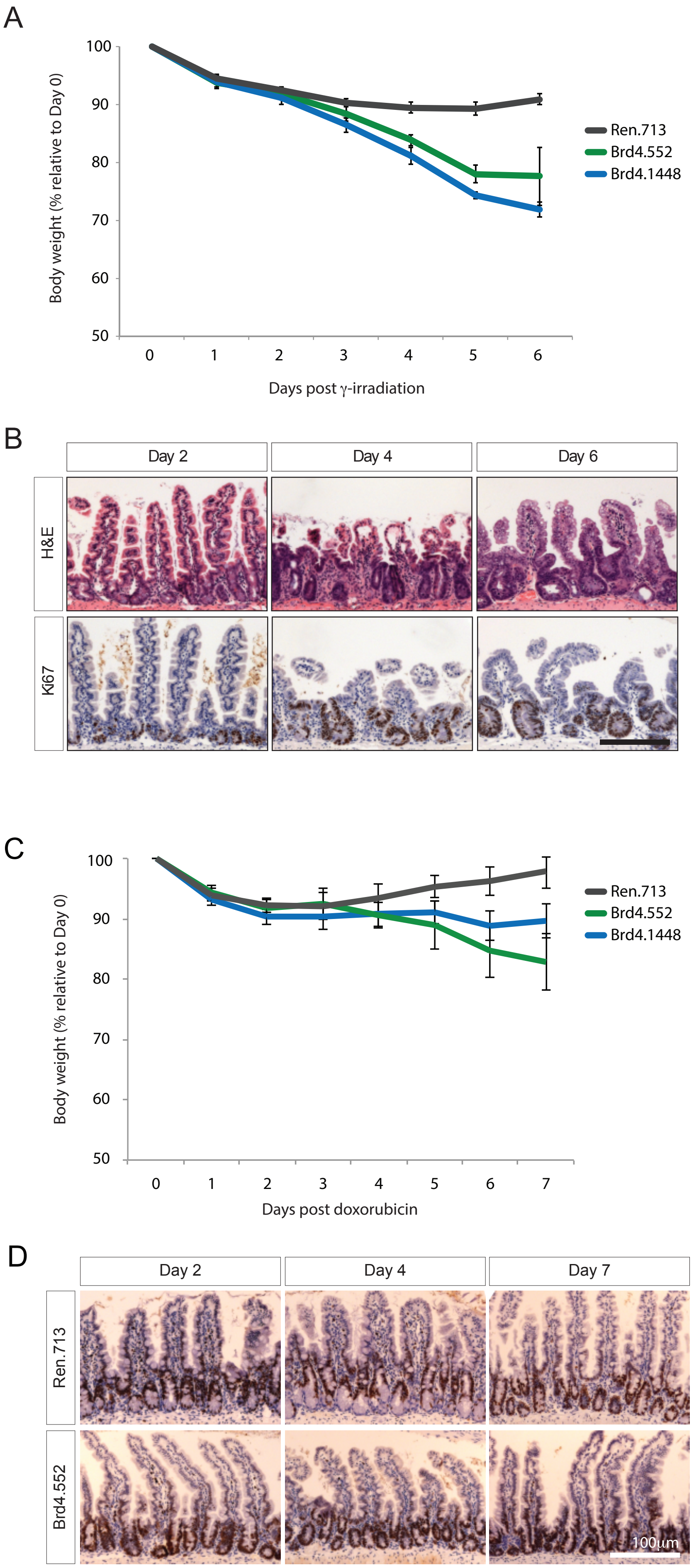
A
Body weight (% relative to Day 0)
100
90
80
70
60
50
0
1
2
3
4
5
6
Days post γ-irradiation
Ren.713
Brd4.552
Brd4.1448
B
Day 2
Day 4
Day 6
H&E
Ki67
C
Body weight (% relative to Day 0)
100
90
80
70
60
50
0
1
2
3
4
5
6
7
Days post doxorubicin
Ren.713
Brd4.552
Brd4.1448
D
Day 2
Day 4
Day 7
Ren.713
Brd4.552
100μm

Table S1. shRNA sequences, related to Figure 1

shRNA	Sequence (*Xho*I/*Eco*RI fragment for miR30-based shRNA cloning, 5'-3')
Brd4.552	CTCGAGAAGGTATATTGCTGTTGACAGTGAGCGCCCCATGGATATGGGAACA<br>ATATAGTGAAGCCACAGATGTATATTGTTCCCATATCCATGGGTTGCCTACTG<br>CCTCGGAATTC
Brd4.1448	CTCGAGAAGGTATATTGCTGTTGACAGTGAGCGACACAATCAAGTCTAAACTA<br>GATAGTGAAGCCACAGATGTATCTAGTTTAGACTTGATTGTGCTGCCTACTGC<br>CTCGGAATTC
Ren.713	CTCGAGAAGGTATATTGCTGTTGACAGTGAGCGCAGGAATTATAATGCTTATC<br>TATAGTGAAGCCACAGATGTATAGATAAGCATTATAATTCCTATGCCTACTGC<br>CTCGGAATTC
Rpa3.455	CTCGAGAAGGTATATTGCTGTTGACAGTGAGCGCGCGACTCCTATAATTTCTA<br>ATTAGTGAAGCCACAGATGTAATTAGAAATTATAGGAGTCGCTTGCCTACTGC<br>CTCGGAATTC
